# Comprehensive Chemical Analysis of the Methyl 3-Nitrogen-2,3-Dideoxysaccharides Derivatives with d-*ribo*-Configuration: Synthesis, Reactivity of HIV-1 Reverse Transcriptase Inhibitors

**DOI:** 10.1021/acs.jpcb.4c08136

**Published:** 2025-01-14

**Authors:** Aleksandra M. Dąbrowska, Rajmund Kaźmierkiewicz, Anna M. Barabaś-Lepak, Małgorzata Biedulska, Agnieszka Chylewska

**Affiliations:** † Intermolecular Interaction Laboratory, Department of Bioinorganic Chemistry, Faculty of Chemistry, University of Gdańsk, Wita Stwosza 63, 80-308 Gdańsk, Poland; ‡ Laboratory of Biomolecular Systems Simulations, Intercollegiate Faculty of Biotechnology, University of Gdańsk and Medical University of Gdańsk, Abrahama 58, 80-307 Gdańsk, Poland; § I Secondary School named after Maria Skłodowska-Curie in Tczew, Maritime School 1, 83-110 Tczew, Poland; ∥ Institute of Biotechnology and Molecular Medicine, Kampinoska 25, 80-180 Gdańsk, Poland

## Abstract

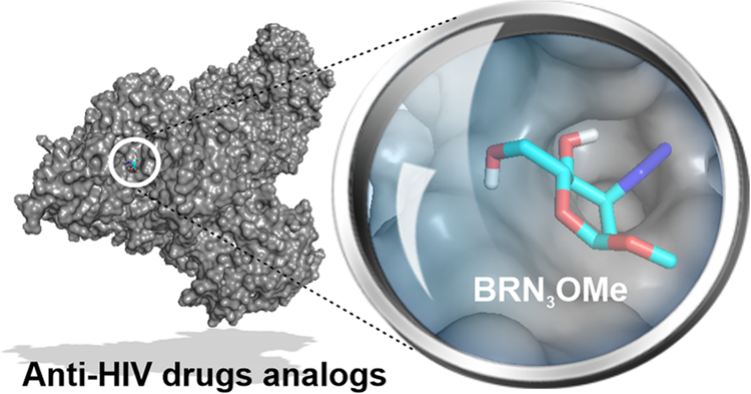

This study extends previous research, particularly focusing on patented scientific objects No. ID: PL 240 353 B1, investigating the physicochemical properties of the methyl 3-azido- and 3-amino-2,3-dideoxysaccharides with a nucleoside scaffold similar to 3′-azidothymidine (AZT). The study utilizes multiwavelength spectrophotometric and potentiometric methods to evaluate the ionization of the saccharide units in aqueous solutions. p*K*_a_ values, obtained from two independent methods, reveal significant sugar ionization effects on UV spectra with varying pH levels. Stability constants for divalent metal ion complexes (Cu^2+^ and Ni^2+^) with the saccharide isomers indicate that complex stoichiometries and stabilities are highly dependent on the configuration of sugar ring substituents. Spectrophotometric results show a descending order of *CT*-DNA-binding affinity: **BRNH**_**2**_**OMe** > **BRN**_**3**_**OMe** > **ARN**_**3**_**OMe** > **ARNH**_**2**_**OMe**, suggesting varied interaction strengths. Molecular docking of models of synthesized *O*-glycosides confirmed their potential as reverse transcriptase inhibitors. Among the derivatives tested, the compound **BRN**_**3**_**OMe** displays the highest interaction with the enzyme active site residues and DNA, suggesting it may possess the greatest efficacy. Our reported results highlight the promising inhibitory properties of novel *O*-glycosides against HIV reverse transcriptase, supporting their potential development as antiviral agents.

## 1 Introduction

Human Immunodeficiency Virus (HIV) continues to pose a significant global health challenge despite substantial advancements in antiretroviral therapies (ART). Current treatments, primarily targeting viral enzymes like reverse transcriptase and protease, have transformed HIV from a fatal disease into a manageable chronic condition.^1,2^ However, issues such as drug resistance, long-term toxicity, and the need for lifelong adherence highlight the necessity for ongoing research and development of novel therapeutic agents. Drug design is an iterative process that commences with a compound exhibiting an interesting biological profile and culminates in the optimization of the molecule’s activity profile and its chemical synthesis.

Azidothymidine (AZT), the initial pharmaceutical agent demonstrated to prolong the lives of individuals afflicted with acquired immune deficiency syndrome (AIDS),^3,4^ necessitates a reevaluation of the testing protocols for this and other potential therapeutic interventions for the disease. AZT exhibits efficacy against HIV exclusively during the replication of the virus into proviral DNA (viral DNA synthesis precedes integration into host DNA). This specificity is attributed to the high affinity of the active compound AZT for a complex of DNA with the enzyme reverse transcriptase (RT), which performs a critical function in viral replication by facilitating the conversion of viral RNA into DNA, thus rendering it a primary target for antiretroviral therapy.^5^ Despite the availability of various RT inhibitors, the emergence of drug-resistant strains requires a continuous search for novel inhibitors with unique mechanisms of action. RT consists of two subunits: p66 and p51.^6^ This enzyme has DNA- and RNA-dependent polymerase of DNA activity and ribonuclease H activity. Subunit p66 is responsible for the activity of polymerase and ribonuclease H, while subunit p51 performs a structural role. The retrovirus uses the HIV-1 reverse transcriptase to transcribe its genetic material from RNA to a DNA molecule which is built up by its integration into host genetic material, which is one of the cells of the human immune system.

Known antiretroviral drugs include azidothymidine (AZT), which inhibits HIV reverse transcriptase and is a nucleotide derivative with a dideoxypyranosyl structure.^7−9^ Another antiretroviral drug, Tenofovir (TFO), also known as (*R*)-9-(2-(phosphanomethoxypropyl)adenine) (PMPA), is a nucleotide analog that inhibits reverse transcriptase.^10−12^ The covalent bond between RT and Tenofovir results in the termination of the DNA primer within the RT complex. Nucleosides that contain the saccharide residue in the form of 2-deoxy-β-d-*ribo*-hexofuranosyl are not well understood. However, due to their similarity to naturally occurring nucleosides of 2-deoxy-β-d-*erythro*-pentofuranosyl (2-deoxy-d-*ribo*-furanosyl), they have become a focus of research interest.^13,14^ AZT closely resembles thymidine, a nucleoside naturally produced by cells and essential for DNA synthesis as a building block of nucleic acids. Unlike thymidine, azidothymidine has a higher affinity for the DNA-reverse transcriptase complex and features an azido group (−N_3_) in place of the typical hydroxyl group (−OH). This structural difference causes reverse transcriptase to incorporate AZT into the elongating strands of HIV proviral DNA. However, the incorporation halts DNA synthesis and replication, as the azido group prevents subsequent nucleosides from binding. One of the promising research directions is the search for saccharide-based derivatives.^15^ These compounds have shown potential due to their structural versatility and the ability to mimic natural substrates of viral enzymes. Among these, the methyl 3-nitrogen saccharide derivatives have shown attention for their promising antiviral properties. Their unique structural features, particularly the introduction of a nitrogen atom at the 3-position^16,17^ are hypothesized to enhance their interaction with viral targets and improve their pharmacological profiles.

In this study, we focus on the synthesis and characterization of a series of the methyl 3-nitrogen saccharide derivatives, designed as analogs of existing anti-HIV drugs. By systematically modifying the saccharide scaffold, we aim to enhance the antiviral efficacy^18,19^ and minimize potential side effects. The derivatives are evaluated for their activity against HIV-1, with a particular focus on their interaction with viral reverse transcriptase.^20^ In the research, we rely on the hypothesis that the presence of nitrogen at the 3-position is responsible for improving the binding affinity to such targets as DNA or the viral enzyme but it also offers better resistance profiles against HIV-1 variants. Through detailed profiling of these compounds, including their: synthesis, structural characterization, and interaction with biomolecules, we aim to present potent candidates for further biological research.^21−24^ The indication of the interaction strength between DNA and the methyl 3-nitrogen saccharide derivatives considers various binding sites and mechanisms, including electrostatic interactions, hydrogen bonds, and sequence specificity. The stability constants of these complexes indicate strong and stable interactions that can modulate the structural and functional properties of DNA. These interactions have potential applications in gene therapy, drug design, and research into DNA structure and function, opening new possibilities in medicine and biotechnology. Moreover, we explore the potential of new sugar derivatives as inhibitors of HIV-1 reverse transcriptase through molecular docking approaches. Molecular docking is a computational method used to determine the most favorable orientation of a ligand when it binds to a protein, offering valuable information about binding affinity and molecular interactions.^25−28^ Simulating the docking of sugar derivatives studied into the active site of HIV-1 RT, we aim to check if new pyranoside scaffolds could serve as leads for the development of effective RT inhibitors.

Interestingly, the p*K*_*a*_ values of aminosugars, reflecting their tendency to protonate or deprotonate, are fundamental to understanding their behavior in biological systems. These p*K*_*a*_ values influence the formation and stability of complexes with metal ions like nickel^29^ and copper.^30^ The stability constants of these complexes highlight the significant affinity between aminosugars and these metal ions, which has important implications for metal ion homeostasis, enzymatic activity, and potential therapeutic applications.^31^ Understanding the nature of these complexes is essential for several reasons. First, it can shed light on the mechanisms underlying copper’s role in biological systems and how its bioavailability and activity are regulated. Second, insights into these interactions could reveal new aspects of carbohydrate chemistry and its applications in bioinorganic chemistry. Lastly, the study of copper-3-aminosugar complexes could lead to the development of novel therapeutic strategies for diseases associated with copper dysregulation, such as Wilson’s disease, Menkes’s disease, and certain neurodegenerative disorders.^32^ Furthermore, the interaction between nickel ions and 3-aminosugar derivatives has garnered increasing attention due to its potential biological implications. Complexes formed between nickel and these sugar derivatives can affect the stability and activity of nickel-dependent enzymes, modulate metal ion transport and storage,^33^ and impact cellular signaling pathways. Moreover, the ability of 3-aminosugars to chelate nickel ions can play a role in detoxification processes and metal homeostasis, ensuring the proper balance of nickel within biological systems.

We believe, that continued research in this area holds the potential to unlock new strategies for treating genetic disorders, cancer, and infectious diseases, underscoring the importance of understanding and harnessing these complex molecular interactions.

## 2 Experimental Section

### 2.1 Chemicals and Precursors

The anomers of the methyl 3-amino-2,3-dideoxy-d-*ribo*-hexopyranosides (**ARNH**_**2**_**OMe**), (**BRNH**_**2**_**OMe**) and their 3-azido precursors (**ARN**_**3**_**OMe**), (**BRN**_**3**_**OMe**) were prepared according to procedures described previously.^15,34^ The aqueous carbohydrate stock solutions were prepared using Merck analytical grade AR chemicals, NaOH (99%), and HCl (37%). Water solvents for copper(II) and nickel(II) chloride were prepared using reagents purchased from Sigma-Aldrich. *Calf Thymus* DNA (*CT*-DNA) (batch No. SLBZ0111) was used in the binding assay without further purification. All the solutions were used for spectrophotometric and potentiometric titrations. The calibration for potentiometric measurements was carried out using standard buffers (pH 4.0 ± 0.01, pH 7.0 ± 0.01, pH 10.0 ± 0.01) at 25 °C, all from Sigma-Aldrich. All solutions were prepared with deionized water from HYDROLAB, free of organic matter.

### 2.2 Measurements and Methods

All ^1^H NMR (700 MHz) and ^13^C NMR (125 MHz) spectra were acquired using Bruker Avance III spectrometer on 298 K. The signal values for the analyzed compounds were obtained in deuterochloroform (CDCl_3_) solutions, with tetramethylsilane (TMS) serving as the internal standard. Infrared spectra were measured using a PerkinElmer 257 spectrophotometer in KBr film. Optical rotations were determined at room temperature with a Hilger and Watts polarimeter, utilizing a 1 dm tube and the sodium D line. Reaction progress was tracked through thin-layer chromatography (TLC) on E. Merck Kieselgel 60 F-254 aluminum plates, employing the following eluent systems (v:v): A, 2:1 EtOAc—*n*-hexane; B, 1:1 EtOAc—*n*-hexane. Column chromatography was conducted using MN Kieselgel 60 (<0.08 mm, E. Merck). Evaporations were performed under reduced pressure at 35–40 °C. UV–vis absorption spectra were recorded on an Evolution 300 double-beam spectrophotometer (Thermo Fischer Scientific, Waltham, MA, USA) equipped with an automatic stirrer and 1 cm quartz cells. The temperature within the cells was strictly maintained using a Lauda EcoLine 003 water bath, type E100, at 25 °C (thermostated). pH measurements were conducted via potentiometric titrations using an automated setup comprising a titration cell, magnetic stirrer, and an automatic titrator fitted with a 0.50 mL Hamilton syringe. The pH electrode (InLab-RoutinePro type) was sourced from Mettler Toledo and calibrated with standard pH buffers.^35^

The binding ability of 3-nitrogen saccharide derivatives with *CT*-DNA was carefully monitored using the same spectrophotometer in the range 200–450 nm with a spectral band of 2.00 nm width using Tris-HCl buffer as a blank. The apparatus is equipped with a magnetic stirrer. The computer-controlled automatic microtitrator CerkoLab, equipped in Hamilton’s syringe (0.50 mL).

### 2.3 Synthesis and Characteristics of the Compounds Undertaken

The synthetic procedures to obtain the all compounds used in our studies were described in.^1,15,34,36^ Resynthesis of all compounds in the necessary amounts was performed again, and the previously uncharacterized structures of the both β-anomers (**BRN**_**3**_**OMe**, **BRNH**_**2**_**OMe**) and the α-anomers of the methyl 3-azide derivative (**ARN**_**3**_**OMe**) are presented below.

#### Methyl 3-Azido-2,3-dideoxy-α-d-ribo-hexopyranoside (**ARN**_**3**_**OMe**)

(35% syrup); other information can be found in^1,36^ IR [cm^–1^]: 3413 ν(OH), 2934, 2836 ν(CH_2_), 2105 ν(N_3_), 1434 δ(CH_2_)+δ(OCH)+δ(CCH); 1374 δ(OCH)+δ(COH)+δ(CCH); 1337 δ(CCH)+δ(OCH); 1191 ν(CO)+ν(CC); 1130 ν(CO); 985 ν(CO)+ν(CC), see Figure S1; ^1^H NMR δ [ppm]: 4.75 (d, 1H, *J*_1,2a_ = 4.3 Hz, H-1); 4.11 (q, 1H, *J*_3,2a_ = 3.5 Hz, *J*_3,4_ = 3.2 Hz, H-3); 3.88–3.83 (m, 3H, H-5, H-6, H-6′); 3.76 dd (1H, *J*_4,5_ = 9.1 Hz, *J*_4,3_ = 3.6 Hz, H-4); 3.39 (s, 3H, OC**H**_**3**_); 2.23 (dd, 1H, *J*_2e,2a_ = 15.1 Hz, *J*_2e,3_ = 2.9 Hz, *J*_2e,1_ = 1.0 Hz, H-2e); 2.04 (dt, 1H, *J*_2a,1_= J_2a,3_ = 4.2 Hz, H-2a); ^13^C NMR δ [ppm]: 96.62 (C-1); 67.77 (C-5); 67.52 (C-6); 62.63 (C-4); 58.27 (C-3); 55.39 (O**C**H_3_); 32.29 (C-2); Figures S2 and S3 with adequate spectra were included in the Supporting Information, see SI file.

#### Methyl 3-Azido-2,3-dideoxy-β-d-ribo-hexopyranoside (**BRN**_**3**_**OMe**)

(35% syrup); *R*_*f*_ 0.28 (solvent A); [α]_D_ −27° (*c* 0,7 methanol); IR [cm^–1^]: 3398 ν(OH), 2933, 2836 ν(CH_2_), 2104 ν(N_3_), 1433 δ(CH_2_)+δ(OCH)+δ(CCH); 1374 δ(OCH)+δ(COH)+δ(CCH); 1337 δ(CCH)+δ(OCH); 1191 ν(CO)+ν(CC); 1131 ν(CO); 985 ν(CO)+ν(CC), see Figure S4; ^1^H NMR δ [ppm]: 4.75 (d, 1H, *J*_1,2a_ = 4.2 Hz, H-1); 4.11 (dd, 1H, *J*_4,3_ = 3.1 Hz, *J*_4,5_ = 7.1 Hz, H-4); 3.89–3.83 (m, 3H, H-3, H-5, H-6); 3.77 (dd, 1H, *J*_6,6′_ = 8.7 Hz, *J*_5,6′_ = 3.5 Hz, H-6′); 3.49 (s, 3H, OC**H**_**3**_); 2.22 (dd, 1H, *J*_2e,2a_ = 14.9 Hz. J_2e,1_ = 0.9 Hz, *J*_2e,3_ = 2.9 Hz, H-2e); 2.03 (dt, lH, *J*_2a,l_ = 4.3 Hz, *J*_2a,3_ = 4.5 Hz, H-2a); ^13^C NMR δ [ppm]: 96.65 (C-1); 67.76 (C-5); 67.48 (C-6); 62.56 (C-4); 58.29 (C-3); 55.39 (O**C**H_3_); 32.3**6** (C-2); Figures S5 and S6.

#### Methyl 3-Amino-2,3-dideoxy-α-d-ribo-hexopyranoside (**ARNH**_**2**_**OMe**)

The synthesis and structural confirmation were published by Liberek et al.^1,36^ The IR and NMR analysis from resynthesis were included in Supporting Information, Figures S7–S9.

#### Methyl 3-Amino-2,3-dideoxy-β-d-ribo-hexopyranoside (**BRNH**_**2**_**OMe**)

(10% syrup); *R*_f_ 0.23 (solvent B); IR: [cm^–1^]: 3352 ν(NH), 2923,2853 ν(CH_2_), 1450 δ(CH_2_)+δ(OCH)+δ(CCH); 1378 δ(OCH)+δ(COH)+δ(CCH); 1210 ν(CO)+ν(CC); 1129 ν(CO); 1070 ν(CO)+ν(CC), see Figure S10; ^1^H NMR δ [ppm]: 4.75 (dd, 1H, *J*_1,2a_ = 8.8 Hz, *J*_1,2e_ = 2.6 Hz, H-1); 3.92 (dd, 1 H, *J*_6,6′_ = 11.7 Hz, *J*_6,5_ = 4.1 Hz, H-6), 3.82 (dd, 1H, *J*_6′,5_ = 5.1 Hz, H-6′); 3.74–3.68 (m, 1H, H-5); 3.63 (dd, 1H, *J*_4,5_ = 8.5 Hz, *J*_4,3_ = 4.2 Hz, H-4); 3.51 (s, 3H, OC**H**_**3**_); 3.47–3.40 (m, 1H, H-3); 1.96 (dq, 1H, *J*_2e,3_ = 2.35 Hz, *J*_2e,1_ = 4.55 Hz, H-2e); 1.80 (dq, 1H, *J*_2a,2e_ = 13,5 Hz, *J*_2a,3_ = 4.4 Hz,, *J*_2a,1_ = 8.9 Hz, H-2a); ^13^C NMR δ [ppm]: 98.76 (C-1); 74.00 (C-5); 68.07(C-4); 63.98 (C-6); 56.57 (O**C**H_3_); 47.50 (C-3); 37.38 (C-2); Figures S11 and S12.

### 2.4 Acidity Constants

The procedure for pH-spectrophotometric determination of acid–base constants has been detailed previously.^37^ Titrations were conducted using 1.00 mM sugar acidic solution (in 0.01 M HCl) as analyte and 0.01 M NaOH serving as titrant. At each pH value, an aliquot of the solution was taken, and its absorption spectrum was recorded. The relationship between maximum absorption and pH was analyzed to calculate the equilibrium constants of the species present in the system. Each experiment was performed in triplicate to ensure reproducibility. All titrations were carried out under full computer automation.

To check the number of dissociation constants for calculation and the number of equilibria in the solution studied, *A*-diagrams and the relationships between absorbance at two different wavelengths were constructed. These diagrams serve as criteria for assessing measurable overlap. Under equilibrium conditions, the diagrams created for all wavelength combinations should exhibit linearity. Importantly, the intersection point of two linear segments on the *A*-diagram indicates the presence of the ampholytic form when two equilibria coexist in the solution. Acidity constant values were determined based on absorbance changes at a chosen wavelength, applying a nonlinear least-squares approach^38^ as described by eq 1:
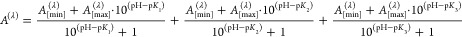
1

This new form of expression was used with *Origin8.6 Software* program to calculate the ionization constants of the compounds studied.

### 2.5 Stability Constants

In a standard procedure, 2.00 mL of the ligand solution in water (analyte) is transferred to the spectrophotometer cell, and the initial absorbance is recorded. Subsequently, a precise volume of a concentrated metal ion solution in the saccharide (prepared at the same concentration as the analyte) is incrementally added using a 1.00 mL Hamilton syringe. Absorbance measurements are taken after each addition. The process is repeated until the target metal-to-ligand molar ratio is attained. The reverse titration method was used in the case of Cu(II) ions complexation with two methyl 3-aminosaccharides because of registered excessively high absorbance.

The formation constants (β*_n_* = Π*K_n_*) and molar absorptivity of the complexes formed between the studied saccharides and M(II) ions at 25 °C were determined by fitting the measured absorbance values (*A*_obs_) to the following equation:
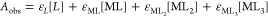
2where ε values are the molar absorptivity of the species denoted. To evaluate the formation constants from the absorbance vs. *C*_L_/*C*_M_ mole ratio data, a nonlinear least-squares Gauss–Newton–Marquardt algorithm was used.^38^ The free metal ion concentration [M] was calculated using the EQUID program.^39,40^ Once the concentration of [M] was determined, the concentrations of all other species were calculated using the mass balance equations and the estimated formation constant at the current interaction step of the program. The EQUID program output included refined parameters, the sum of least-squares, and the standard deviation of the data.^39,40^ The adjustable parameters comprised the stepwise formation constants for all complexes present and their associated molar absorptivities.

The ChemAxon MARVIN software^41^ was used to predict the total charge distribution of the four the methyl 3-nitrosugar molecules.

### 2.6 Methodology of Binding Ability Assay of the Methyl 3-Nitrogen Saccharide Derivatives with *CT*-DNA

The electronic absorption titrations were carried out to determine the type and strength of 3-nitrogen saccharide derivatives intermolecular interactions with *Calf Thymus* DNA (*CT*-DNA). The stock *CT*-DNA solution was dissolved in working buffer pH 7.32 (5.00 mM Tris-HCl, 50.00 mM NaCl). The purity and concentration of DNA were determined spectrophotometrically. The ratio of absorbance at 260 and 280 nm was 1.92, indicating that the *CT*-DNA applied in the binding assay was not contaminated by proteins.^42^ The computer-controlled automatic micro-titrator CerkoLab was used to introduce a precise volume of 0.0042 mL of *CT*-DNA in each step of the measurement. The final concentration of the prepared *CT*-DNA stock solution to be 101.48 mM was determined by measuring its absorbance at 260 nm and applying a molar extinction coefficient (ε) of 6600 M^–1^ cm^–1^.^43^

The electronic absorption titrations were carried out in a 1.00 cm quartz cuvette at a fixed 3-nitrogen saccharide derivatives in aqueous media at room temperature by varying the concentration of nucleic acid in the range 200–450 nm. The stock solutions of saccharide derivatives were prepared in 5.00 mM Tris-HCl buffer pH 7.32 and their concentrations were as follows: [**ARN**_**3**_**OMe**] = 6.44 mM; [**BRN**_**3**_**OMe**] = 0.85 mM; [**ARNH**_**2**_**OMe**] = 3.41 mM; [**BRNH**_**2**_**OMe**] = 4.92 mM. After each addition of *CT*-DNA, the solution of the appropriate saccharide derivative was mixed well, and the electronic absorption spectra of specific formed adducts were recorded in the range of 200–450 nm. With increasing concentration of nucleic acids in the 3-nitrogen saccharide derivative solution, the increase in absorbance and band shifts were carefully monitored until full binding was achieved. It should be emphasized that the dilution effects related to the measurement protocol were eliminated at the initial step of developing the results.

Based on absorbance variability, the intrinsic binding constant, *K*_bind_ description strength of the interaction of the methyl 3-nitrogen saccharide derivatives with *Calf Thymus* DNA was determined using the Benesi–Hildebrand equation:^44^
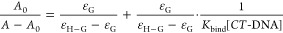
3where *A*_0_ and *A* are the absorbance values of the free 3-nitrogen saccharide derivatives and the sugar—DNA complex, respectively. The molar absorption coefficient corresponding to the unbound sugar derivative is denoted as ε_*G*_, whereas ε_H-G_ corresponds to the saccharide—*CT*-DNA adduct. Consequently, the double reciprocal plot of *A*_0_/(*A* – *A*_0_) against 1/[DNA] exhibited linearity, and the binding constant values were determined from the ratio of the y-intercept to the slope using linear regression parameters.

### 2.7 Molecular Docking

For docking experiments we used the crystal structure of HIV-1 reverse transcriptase structure deposited in PDB database with ID: 1T03. This structure was chosen due to the following reasons: (i) it is a completed structure of reverse transcriptase with no missing atom coordinates, (ii) it contains an original sequence of RT of the human HIV-1 virus, which means that there are no mutations in its sequence. It also contains a nucleic acid fragment consisting of a template strand with the following nucleotide sequence: 5′-ATGCATGGCGCCCGAACAGGGACTGTG-3′ and a primer strand with nucleotide sequence: 5′-ACAGTCCCTGTTCGGGCGCC-3′. The presence of nucleic acid fragments in this structure is necessary for compound analysis, as the enzyme without nucleic acid would not be able to perform its biological function. Additionally, the presence of nucleic acid fragments, as well as the presence of an inhibitor in the enzyme’s active site (the X-ray structure has an inhibitor–tenofovir) ensures that this is an active form of the enzyme. The presence of the inhibitor in the active site provides an insight into where exactly this active region is located (without the need to search for it), which is very favorable, because, in general, the specific location of the active center in a given protein is not clear. This structure was first described in a high-profile science magazine.^45^ Molecular docking was performed using Autodock Vina, involving the search for 44,000 low-energy complex configurations of HIV reverse transcriptase with each synthesized AZT analog, with AZT also docked for comparison. In total, 220,000 models of complex structures were obtained. For each of the four synthesized AZT analogs, the lowest energy conformations were identified. These conformations were then clustered into families with similar arrangements using the Gromacs rms tool. One conformation of the complex for each deoxysaccharide derivative and AZT was selected for more detailed analysis.

## 3 Results and Discussion

**Figure 1:**
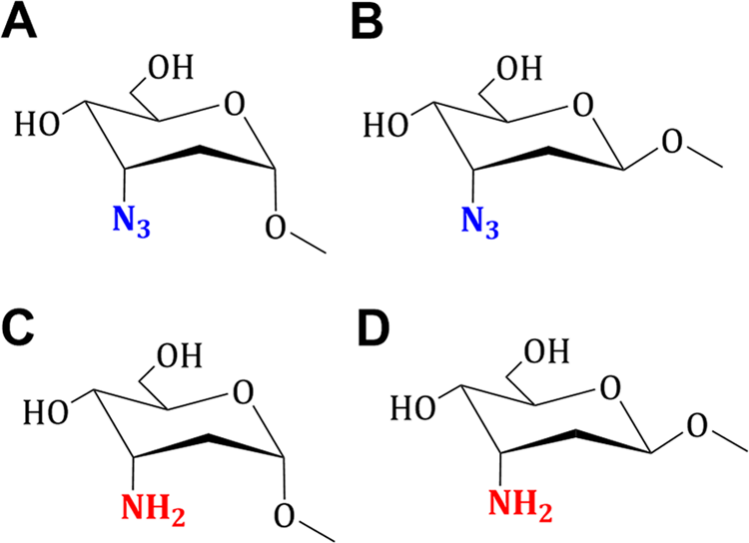
Structures of the investigated sugar molecules were named **A**. ARN_3_OMe, **B**. BRN_3_OMe, **C**. ARNH_2_OMe, and **D**. BRNH_2_OMe.

Figure 1 presents structures of the four investigated molecules named anomers of the methyl 3-azido-2,3-dideoxyhexopyranosides with α-d-*ribo*, and β-d-*ribo* configurations, and their 3-amino derivatives, respectively. The acronyms were introduced later in the text for better clarity: **ARN**_**3**_**OMe** for the methyl 3-azido-2,3-dideoxy-α-d-*ribo-*hexopyranoside, **BRN**_**3**_**OMe** for the methyl 3-azido-2,3-dideoxy-β-d-*ribo-*hexopyranoside, **ARNH**_**2**_**OMe** for the methyl 3-amino-2,3-dideoxy-α-d-*ribo-*hexopyranoside, and **BRNH**_**2**_**OMe** for the methyl 3-amino-2,3-dideoxy-β-d-*ribo-*hexopyranoside.

### 3.1 pH Influence

All water solutions of the methyl *O*-glycosides were colorless; therefore, the absorption spectra were prepared in the range of 220–400 nm. The methyl 3-azide anomers were characterized by two absorption bands at 282 and 272 nm for **ARN**_**3**_**OMe** and 286 and 266 nm for **BRN**_**3**_**OMe**. All the observed bands correspond to π → π* transitions, and n → π* may overlap with the stronger π → π* transitions. Substitution by electronegative groups at the C-3 position lowers the energy of electrons on the nitrogen bonded to carbon via induction, resulting in a redshift of the two transitions. Thus, the 3-amine derivatives are characterized by three absorption bands at 350, 314, and 288 nm in the case of **ARNH**_**2**_**OMe** and 306, 292, and 268 nm in the case of **BRNH**_**2**_**OMe**. The pH of the sample solution also significantly affected the absorption spectra. These relationships are presented in Figure S13 in the SI file.

It was observed slight effect of pH on changes in the shift of the maximum absorption of the band from the methyl 3-azide derivatives and larger differences in the case of the amine saccharides. Due to the protonation of the amine a blue shift (hypsochromic shift) was detected, along with an increase in intensity. Diagrams of absorbance vs. concentration were prepared. The relationships for all investigated solvents were linear in the measured range. It was consistent with Beer–Lambert law. The molar absorption coefficients (ε) of the saccharides are presented in Table 1.

**Table 1:** Molar Extinction Coefficients (ε [dm^3^·mol^–1^·cm^–1^]) in the Studied Compounds Solutions of Different pH Medium Determined at 25 °C; the Standard Deviations Computed Refer Only to Random Errors

compound	HCl_(aq)_	H_2_O	NaOH_(aq)_
ARN3OMe	270 nm	270 nm	272 nm
	42.90 (±0.28)	38.99 (±0.42)	33.49 (±0.07)
BRN3OMe	266 nm	266 nm	264 nm
	400.45 (±4.21)	384.25 (±6.59)	145.55 (±2.43)
ARNH2OMe	306 nm	314 nm	288 nm
	63.57 (±4.01)	26.42 (±0.75)	50.19 (±1.11)
BRNH2OMe	292 nm	289 nm	306 nm
	81.54 (±0.29)	68.67 (±0.89)	66.79 (±0.59)

Small values of molar absorption coefficients are correlated with the relatively high concentrations of saccharide solutions used and low absorbance. The shapes of the absorption spectra in neutral, acidic, and basic solutions are different despite their similar concentrations (see Figure S13); however, an increase of intensity of the maximum can be observed in the case of lowering pH. In the case of the methyl 3-azido saccharides the maximum band shifts to higher wave values in the basic environment, and for the methyl 3-aminosaccharides the maximum shifts to a lower wavelength. Particularly noteworthy is the fact that **BRN**_**3**_**OMe** has a molar absorption coefficient almost ten times higher than other compounds.

### 3.2 Ionization Constants

**Figure 2:**
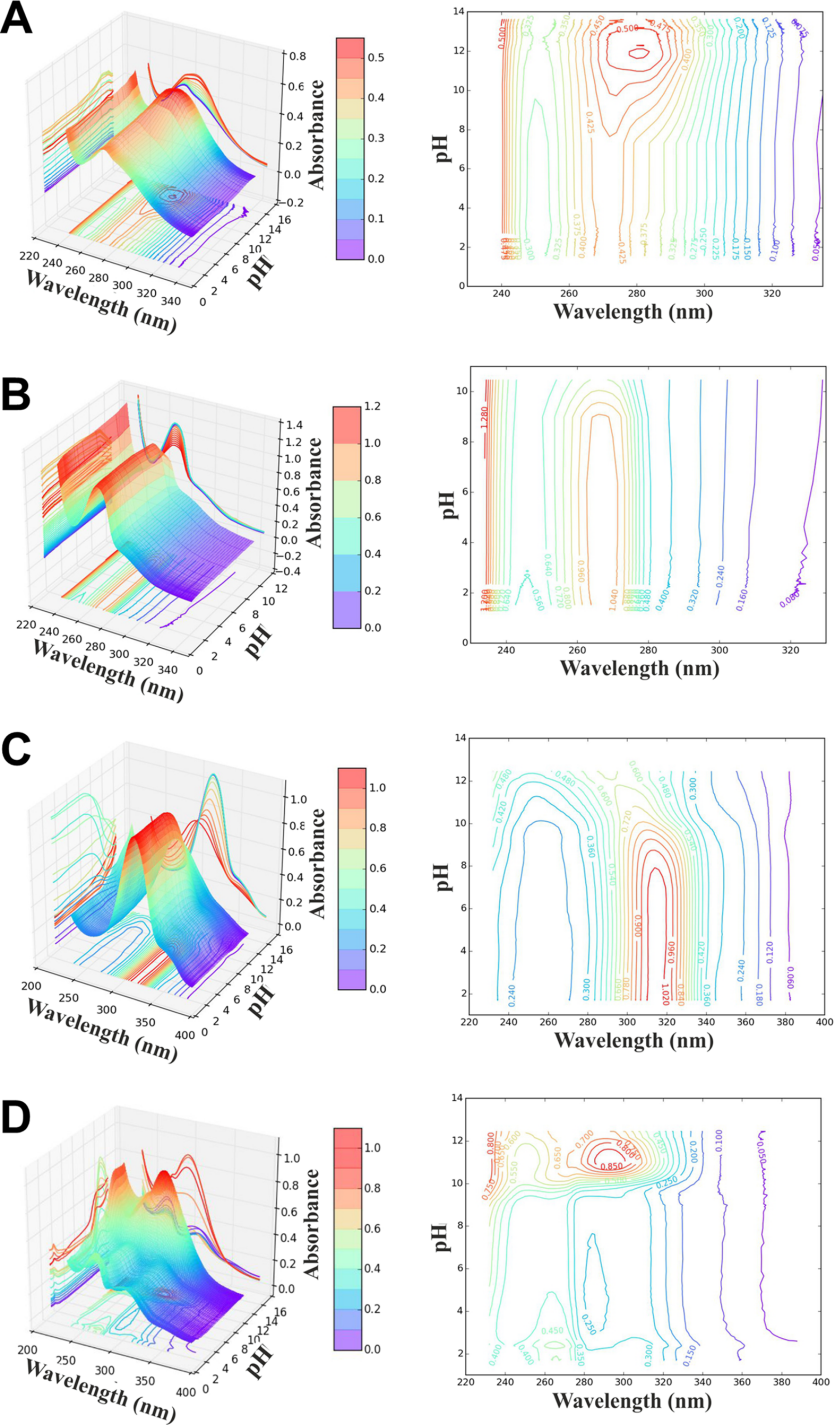
3D-Plots of absorption spectra (left side) of **A**. ARN_3_OMe; **B**. BRN_3_OMe, **C**. ARNH_2_OMe; and **D**. BRNH_2_OMe as the result of pH-spectrophotometric titration; the results of contour map (right side).

The characteristic features of the absorption spectra of organic compounds with acidic or basic substituents are usually pH-dependent. Both the shape and intensity of the absorption bands change as the hydrogen ion concentration varies. The principle of the spectrophotometric method for the determination of acidity or basicity constants relies on surveying changes in the absorption spectra under the influence of varying the pH of the medium. The UV spectra of the two pairs of sugar anomers in aqueous solutions at various pH values are shown in Figure S14. The presence of isosbestic points in the resulting spectra suggests that more than two species are in equilibrium under the experimental conditions. In agreement with the calculated protonation sites,^46,47^ more than three acidity constants associated with the proton-transfer process can exist. The p*K* values of the studied compounds were evaluated pH-spectrophotometrically in aqueous solution at 25 °C. To calculate the respective equilibrium constants, the concentration ratios of neutral to protonated species or deprotonated to neutral species were determined. Consistent conditions used for pH measurements were maintained throughout the process. The experimental data for the studied compounds are shown in Figure 2 as three-dimensional (3D) plots illustrating the relationships between absorption, wavelength, and pH. For a one-step system, the *A*-diagram remains linear if the absorbance at any wavelength is proportional to that at any other wavelength. However, when two or more equilibria govern a system, the *A*-diagrams will change direction each time a new equilibrium becomes dominant.^48^ These diagrams are especially useful for systems where the spectra alone do not reveal the number of equilibria. The corresponding selected *A*-diagrams for three compounds, **ARN**_**3**_**OMe**—Figure S15(A), **BRN**_**3**_**OMe**—Figure S16(A), and **BRNH**_**2**_**OMe**—Figure S18(A) in SI, demonstrate the presence of three distinct linear segments for different wavelength combinations within the studied region. Consequently, based on these *A*-diagram data, it was concluded that for these compounds, three equilibria existed in the studied systems. However, the *A*-diagram for **ARNH**_**2**_**OMe** shows four linear segments (Figure S17(A) in the SI), which indicates the presence of one more (compared with other isomers) equilibrium in the solution of this compound.

The pH-spectrophotometric titrations method provides important information about the amount of species formed during the deprotonation process. The data from a potentiometric titration (hybrid titration) can also be represented as a series of titration curves, showing absorbance as a function of pH (see Figures S15(B)–S18(B) in SI). During acid–base titration, changes in the wavelength versus the pH range are strictly dependent on the type of compound investigated (Figure 2).

In the case of the methyl 3-amino derivatives, we observed the appearance of a new maximum absorption band due to the amine proton acceptor group (Figure 2C,D). During deprotonation, the conjugated acid of the amine derivatives **ARNH**_**2**_**OMe**, **BRNH**_**2**_**OMe** the bands move to the red region. This process occurs in nonacidic environments, as in the case of azido compounds, so the spectrum shows a few isosbestic points and more proton-moving equilibrium, perhaps as a result of intramolecular hydrogen bonds. The calculation aimed at determining the p*K* values was based on the exponential form of the Henderson–Hasselbalch eq 1.^48^ The resulting average p*K* values for all the investigated compounds and the corresponding standard deviations are summarized in Table 2.

**Table 2:** Experimental Ionization Constants of the Studied Compounds were Determined by Spectrophotometric and Potentiometric Methods at 25 °C; the Computed Standard Deviations Refer Only to Random Errors

method	compound	p*K*_1_	p*K*_2_	p*K*_3_
pH-spectro-photometric	ARN_3_OMe	4.11 (±0.12)	10.21 (±0.09)	13.52 (±0.08)
	BRN_3_OMe	1.79 (±0.08)	9.43 (±0.14)	11.08 (±0.08)
	ARNH_2_OMe	3.18 (±0.07)	9.85 (±0.02)	12.29 (±0.07)
	BRNH_2_OMe	4.26 (±0.05)	9.26 (±0.90)	11.01 (±0.04)
potentiometric	ARN_3_OMe	2.94 (±0.90)	7.37 (±0.40)	11.87 (±0.60)
	BRN_3_OMe	4.01 (±0.22)	11.46 (±0.28)	12.47 (±0.64)
	ARNH_2_OMe	4.59 (±0.08)	9.93 (±0.12)	10.32 (±0.18)
	BRNH_2_OMe	7.70 (±0.06)	9.04 (±0.08)	12.33 (±0.13)

Potentiometric data were analyzed using a program developed in our laboratory. This computer program was able to assess the protonation constants under highly acidic conditions, thereby offering a more accurate fit for the potentiometric data. All obtained data are presented as potentiometric titration curves–experimental points and best-fit line and corresponding microspecies distribution (%) diagrams presented at the given pH (Figures S19(B)–S22(B) in SI). The sugar molecules under study possess specific functional groups that are prone to losing (hydroxyl) or gaining (azido and amine) protons under certain experimental conditions. Each equilibrium between the protonated and deprotonated forms of the molecule is characterized by a constant value known as p*K* as presented in Table 2.

The p*K* values were calculated for all the molecules investigated based on their concentration distributions. Figures S19(B)–S22(B) in the SI show the pH-dependent diagrams of the microspecies. The p*K* values are related to the different chemical structures of all compounds studied. The neutral forms of sugar derivatives are weak acids because they possess hydroxy-, methoxy, and azido (**ARN**_**3**_**OMe**, **BRN**_**3**_**OMe**) or amine (**ARNH**_**2**_**OMe**, **BRNH**_**2**_**OMe**) groups.

**Figure 3:**
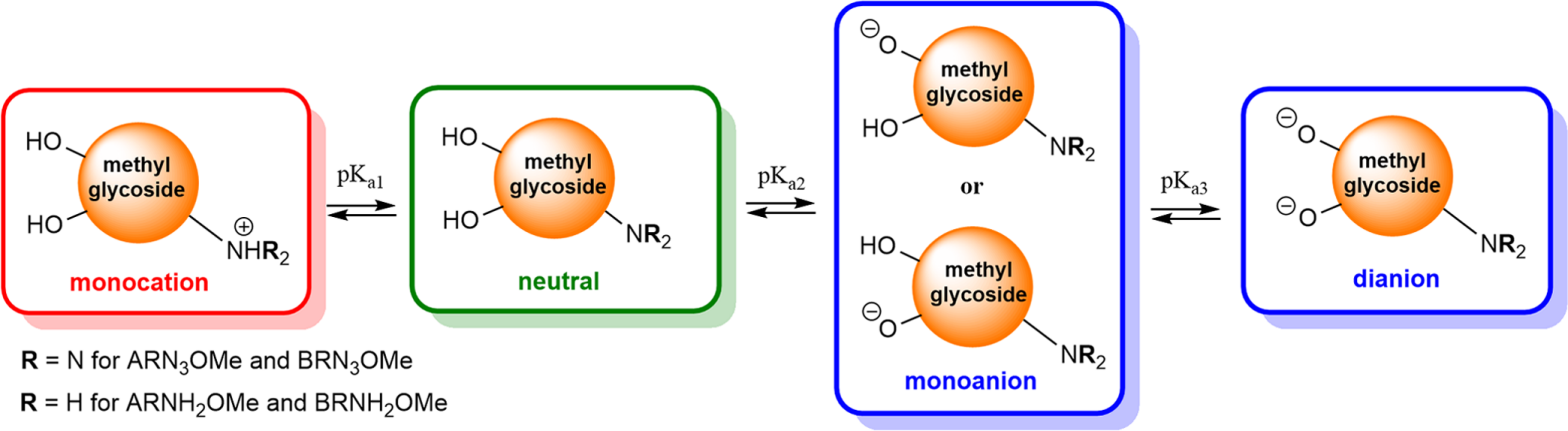
Schematic model of equilibria in aqueous solutions for the title compounds.

Their conjugated acids are stronger because the more acidic protonated azido group (R-N_3_H^+^, where R denotes sugar derivative residue) or protonated amino group (R-NH_3_^+^, where R denotes sugar derivative residue) are inactive, while less acidic hydroxyl groups are available. The predominant microspecies under physiological conditions (pH 7.40) were the neutral forms of all the studied compounds with azido, amine, and hydroxyl sites. The suggested model of deprotonation reactions that occurred in aqueous solutions of all the studied compounds is presented in Figure 3.

Previously, we reported our results obtained from quantum-mechanical calculations in the gas phase, considering the solvation effects for methyl 3-azido-6-iodo-2,3,6-trideoxy-α-d-*arabino*-hexopyranoside^37^ and four isomers of methyl 3-azido-2,3-dideoxy-d-hexopyranosides^46,49^ and their 3-amino derivatives.^47,50^

The results of the calculations performed for the nine isomers, considering plausible protonation centers, led us to conclude that in all isomers, the preferred protonation site is the nitrogen atom of the azido (or amine) group located near the pyranoside ring. The deprotonation energy of these compounds indicates that the primary hydroxyl group is more reactive than the secondary one. Our previous studies^37,46,50^ have shown that both protonation and deprotonation reactions are strongly influenced by the configuration of the anomeric center, solvent nucleophilicity, and steric effects arising from the spatial relationships between substituents in the pyranoside ring. The evaluation of the ionization constants of the methyl 3-azido-6-iodo-2,3,6-trideoxy-α-d-*arabino-*hexopyranoside^37^ showed that acceptable p*K* experimental values could be obtained, which is in agreement with the deprotonation equilibria calculated using theoretical methods.

Analysis of the ionization constant values suggests that the exchange configuration of the substituent at the anomeric center in the same type of 2,3-dideoxysugar derivatives affects the p*K* values, supporting our earlier findings.^37,46,50^

### 3.3 Stability Constants

Absorption bands of aqueous solutions of Cu(II) and Ni(II) ions occurred in the visible region (Figure S23 in the SI), respectively.

The band appearing in the absorption spectrum of paramagnetic copper(II) at 590–615 nm is assigned to the ^2^*E*_g_ → ^2^*T*_2g_ transition in a distorted tetrahedral geometry.^51^ The diamagnetic nature of the nickel(II) complexes and the appearance of bands at 650–730 and 400 nm were assigned to ^3^A_2g_ → ^3^T_2g_, ^3^A_2g_ → ^3^T_1g_(F), and ^3^A_2g_ → ^3^T_1g_(P) transitions in the square planar environment around nickel with *D*_4*h*_ symmetry. If the distance between the sublevels, due to the effect of the electrostatic field of the ligands, increases, the bands corresponding to these transitions appear to shift to shorter wavelengths. The increase in the covalent character of the bonds results in a shift in a similar direction; furthermore, the intensity of the bands increases with the σ-donor properties of the ligand.^52^

**Figure 4:**
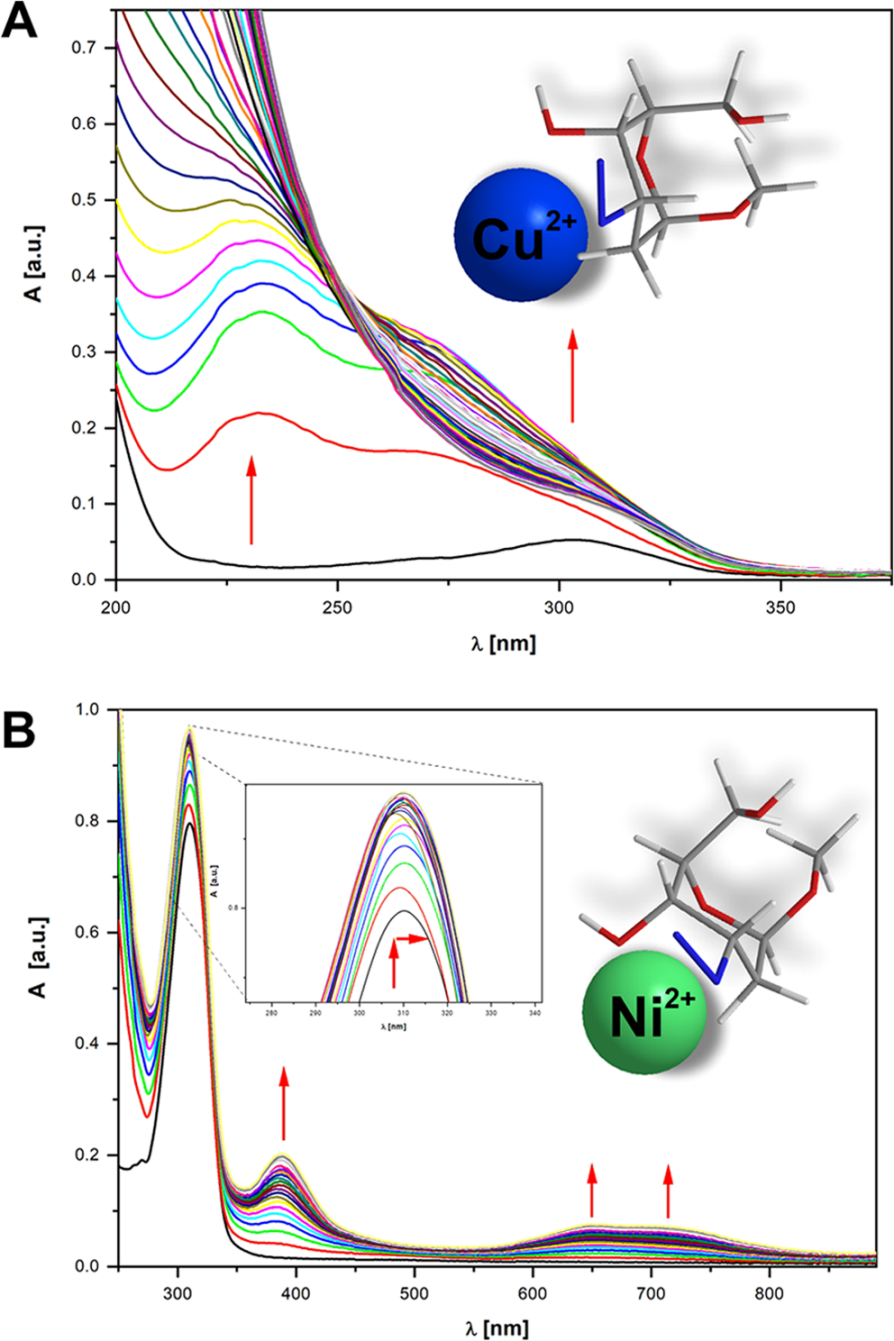
Changes in the electronic absorption spectra during the complexation of BRN_3_OMe with **A**. CuCl_2_ and **B**. NiCl_2_ in aqueous medium at 298 K (arrows indicate different chromic shifts observed).

The methyl 3-azido saccharides **ARN**_**3**_**OMe** and **BRN**_**3**_**OMe** interacted complexometrically with Cu^2+^ or Ni^2+^ ions, showing changes in absorption positions and intensities of bands during spectrophotometric registration (Figures S24 and S25 in the SI). The representative complexometric titration curves for **BRN**_**3**_**OMe** derivative binding of both divalent ions are presented in Figure 4.

The electronic absorption spectrum of NiCl_2_ contains bands with maxima at 400 ± 2 nm (ε = 4.86) and 730 ± 2 nm (ε = 1.97). No shift in the first band (400 ± 2 nm) was observed in the electron absorption spectra of the solution obtained by the addition of the methyl 3-azido compounds to an aqueous solution of NiCl_2_. The second absorption band (657 ± 2 nm) exhibited a slight absorbance that was neglected in the measurable range. The same effect was observed for Cu(II) complexation with **ARN**_**3**_**OMe** and **BRN**_**3**_**OMe**. Determining the stability constants of complexes with the methyl 3-azido saccharides as ligands was possible in the range of 220–400 nm. The addition of Cu(II) ions to an aqueous solution of **ARNH**_**2**_**OMe** or **BRNH**_**2**_**OMe** induced significant changes in the absorption spectra.

The alterations in the absorption spectra of **ARNH**_**2**_**OMe and BRNH**_**2**_**OMe** can be ascribed to the formation of donor–acceptor complexes involving Cu^2+^ ions, with the participation of the vicinal amine and hydroxyl groups. Intensity changes in the whole wavelength region and the new bands appearing (below 300 nm) were observed for both **ARNH**_**2**_**OMe** and **BRNH**_**2**_**OMe** as a result of complexation with both divalent metal ions.

Complex formation was investigated at identical concentrations of all compounds across a broad range of metal ion concentrations. The low and constant concentration of the electroneutral ligand ensured that the theoretical possibility of forming two-ligand complexes could be excluded. Information about the stoichiometry of the metal–ligand complexes was obtained using the continuous variation method.^39,45^ Based on spectrophotometric titration curves (*i.e*., plots of A vs. [M^2+^]) (Figure S26), the stability constants were calculated and are presented in Table 3 (for complexes with Cu^2+^ and Ni^2+^). The formation constants for chelating complexes of all the methyl 3-nitro saccharides with Cu(II) were higher than those obtained for complexes with Ni(II).

**Table 3:** Stability Constants (log β) for Cu^2+^ or Ni^2+^ Complexes with the Studied Compounds and Standard Deviation Obtained from Experimental Data^a^

Cu(II) complexes with	log β_1_	log β_2_	log β_3_
ARN_3_OMe	3.22 (±0.08)	4.12 (±0.33)	6.19 (±0.40)
BRN_3_OMe	2.23 (±0.58)	4.82 (±0.46)	7.57 (±0.68)
ARNH_2_OMe	4.89 (±0.82)	7.89 (±0.34)	11.11 (±0.25)
BRNH_2_OMe	5.79 (±1.65)	8.28 (±1.34)	10.84 (±1.50)

Ni(II) complexes with	log β_1_	log β_2_	log β_3_
ARN_3_OMe	1.51 (±0.80)	3.37 (±0.44)	5.39 (±0.56)
BRN_3_OMe	2.12 (±0.93)	4.74 (±0.66)	7.52 (±0.65)
ARNH_2_OMe	3.37 (±0.88)	4.85 (±0.12)	6.06 (±0.16)
BRNH_2_OMe	2.02 (±1.79)	4.37 (±1.82)	7.03 (±2.82)

a These values were calculated using the EQUID program.

Likely, the different behavior of the azido and amino groups relative to both metal ions expressed in higher or lower stability constants could be explained by the appropriate basicity of both nitrogen atoms. Basicity, however, is not the only factor to be considered when choosing a ligand, but also nucleophilicity. 3-Amine derivatives should be better ligands than their bridging ligand-like azido analogs because of the softer character of the *N*-atom in this group, which corresponds with the soft character of Ni(II), whereas the *N*-atom in the azido group is harder and therefore fits better with Cu(II).

It is important to note that in the spectrophotometric method, the inputting of metal cations to ligands leads to the formation of ML*_n_* complexes initially, due to the high concentration of ligand, followed by the formation of ML_(*n*–*x*)_ complexes at higher metal cation-to-ligand mole ratios. The complexes with lower ligand content are more stable; therefore, the mole ratio plots indicate weak curvature at 1:2 (Figure S26B) and strong curvature at 1:1 (Figure S26A) metal cation-to-ligand mole ratios. Samples of the resulting plots with *A*-diagrams are shown in Figure S27, which suggests that 1:1, 1:2, and 1:3 (metal ion to ligand) complexes could formed in the aqueous solution. As shown in Figures S24 and S25, no sharp isosbestic points were observed in the absorption spectra of the studied ligands in the presence of Cu^2+^ and Ni^2+^, indicating that there is more than one species in equilibrium in the solution. Nickel ions have a lower tendency than other first-row transition metal ions to exchange water molecules for halogen ligands.^53^ This is likely due to ligand field stabilization in octahedral geometries for Ni(II).^51^ Chloride ions generate a weaker ligand field compared to the water dipole, leading to destabilization, which is most pronounced in the case of Ni^2+^. The azido ions as pseudohalogen ions generate a stronger ligand field than that of chloride ions but are still significantly weaker than that of the water dipole. As a result, Ni^2+^ exhibits a stronger preference for water over azido ions. However, at higher azide ion to nickel ion molar ratios, the replacement of more than one water molecule as a ligand can be observed and complexes such as [Ni(N_3_)_2_(H_2_O)_4_], may be occurred. However, the formation of azido-rich complexes was not expected at the ratios used in our experimental studies.

The proposed models of equilibria derived based on the stereochemistry of the saccharide, number of substituents in the ring, and calculated data are presented in Table 4.

**Table 4:** Models of Equilibria were Calculated by the EQUID Computer Program Adapted to Analyze the Spectrophotometric Data (Ion Charges were Excluded for Simplicity)

model	equilibrium	stability constants relationship
1°	M + L = ML	*K*_1_ = β_1_ = [ML]/[M][L]
2°	M + 2L = ML_2_	β_2_ = [ML_2_]/[M][L]^2^
3°	M + 3L = ML_3_	β_3_ = [ML_3_]/[M][L]^3^

The number of straight lines in the *A*-diagrams suggests the existence of at least three possible equilibria.^54^ During classical titration, the concentration of metal ions increased, which could have caused an increase in the absorbance of the saccharide. The rise in the curve can be partially caused by the equilibria and the aforementioned effect. Table 4 lists the calculated values of the stability constants, presented as log β and standard deviation for the examined compounds.

### 3.4 Atomic Charge Distributions

Distribution maps are graphical representations of such properties of molecules as

(a) atomic charge (to analyze the distribution of electron density, highlighting areas of partial positive or negative charges),
(b) electron density (useful for predicting areas of nucleophilic or electrophilic attacks in molecule helping to understand its reactivity),
(c) electrostatic potential (help identify sites of in interactions like hydrogen bonding, dipole–dipole interactions, or electrostatic attractions),
(d) partial charges on individual atoms or groups within a molecule (reflecting the uneven distribution of electron density due to differences in electronegativity).^55,56^ These distribution maps are valuable tools for understanding molecular behavior, predicting reaction mechanisms, identifying reactive sites, and explaining molecular interactions.

All compounds under investigation contained hydroxyl groups as well as azido (**ARN**_**3**_**OMe** and **BRN**_**3**_**OMe**) and amine (**ARNH**_**2**_**OMe** and **BRNH**_**2**_**OMe**) functional groups, which are prone to gaining or losing protons under specific conditions. The chemical bonds between atoms within a sugar molecule involve one or more electron pairs shared between connected atoms. These bonding electrons are unevenly distributed among atoms with differing electronegativities.

**Figure 5:**
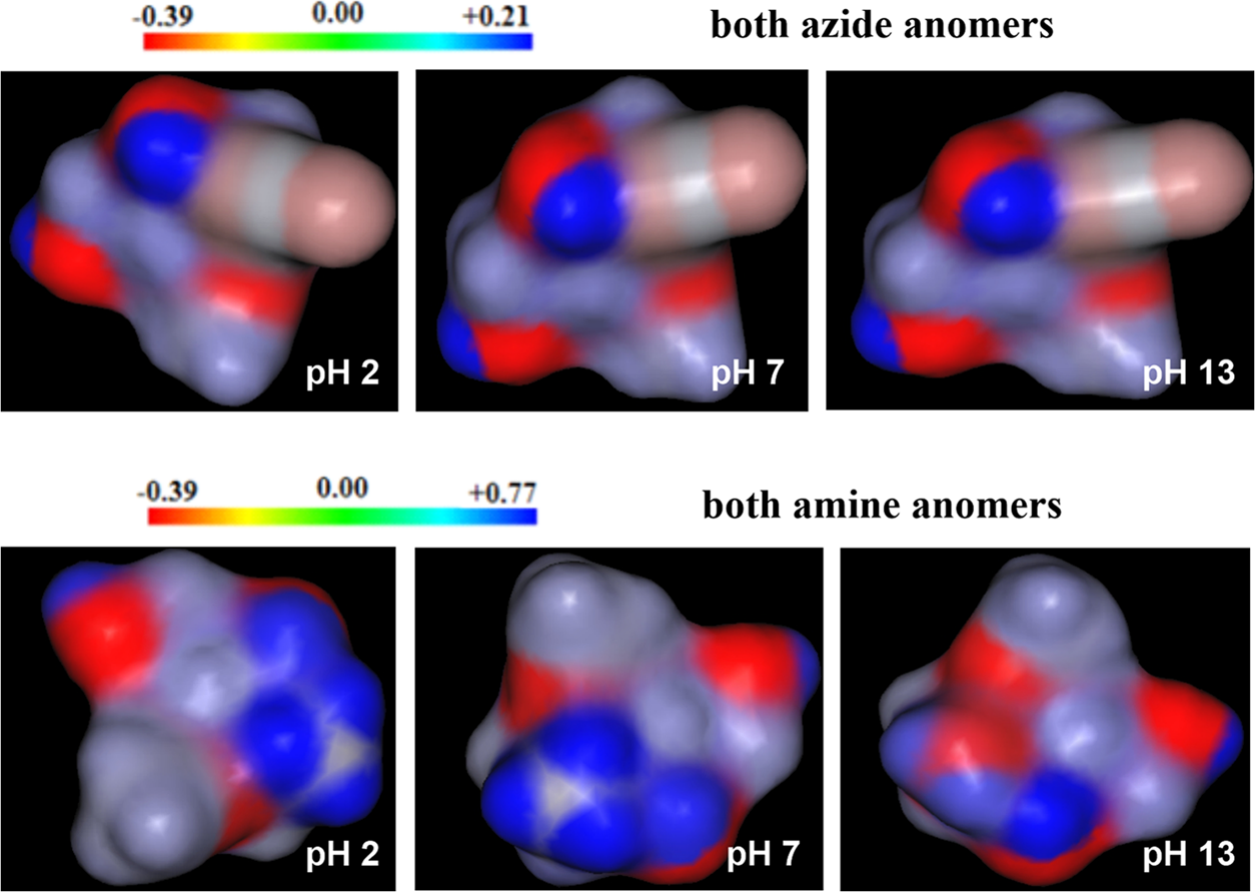
Visual representation of the chemically active sites in major microspecies of the methyl 3-azido-2,3-dideoxy-d-*ribo-*hexopyranoside anomers (top) and the methyl 3-amino-2,3-dideoxy-d-*ribo-*hexopyranoside anomers (bottom) predicted by MARVIN software.

The partial charge distribution helps predict the reactivity of each atom, which is a member of the pyranoside ring. Electron-donating substituents increase the partial charge, in contrast to electron-withdrawing groups, which decrease the partial charge. The MARVIN software was used to predict the charge distributions of the studied sugar molecules. Figure 5 provides a visual representation of the chemically active sites, highlighting the negative (red) regions linked to electrophilic reactivity and the positive (blue) regions associated with nucleophilic reactivity. This density map is an approximate distribution (the same for pairs of anomers) because it does not include the effects of configuration changes or the size of the substituents.

The total atomic charges of all compounds presented as schematic structure in Figure S28 (included in SI) were calculated from σ and π charge components listed in Table 5.

**Table 5:** Total Atomic Charges of All Compounds were Calculated Using the MARVIN Software

	charge (**ARN**_**3**_**OMe** or **BRN**_**3**_**OMe**)			charge (**ARNH**_**2**_**OMe** or **BRNH**_**2**_**OMe**)		
atoms^a^	pH 2	pH 7	pH 13	pH 2	pH 7	pH 13
C(1)	0.16	0.16	0.16	0.16	0.16	0.16
C(2)	0.02	0.02	0.02	0.05	0.05	0.02
C(3)	0.12	0.12	0.12	0.03	0.03	0.04
C(4)	0.10	0.10	0.10	0.13	0.13	0.10
C(5)	0.11	0.11	0.11	0.12	0.12	0.11
C(6)	0.07	0.07	0.07	0.07	0.07	0.07
C(7)	0.04	0.04	0.04	0.04	0.04	0.04
H(1)	0.09	0.09	0.09	0.09	0.09	0.09
H(2)	0.07	0.07	0.07	0.08	0.08	0.07
H(3)	0.05	0.05	0.05	0.10	0.10	0.05
H(4)	0.06	0.06	0.06	0.07	0.07	0.06
H(5)	0.07	0.07	0.07	0.07	0.07	0.07
H(6)	0.12	0.12	0.12	0.12	0.12	0.12
H(7)	0.16	0.16	0.16	0.16	0.16	0.16
O(*endo*)	–0.34	–0.34	–0.34	–0.34	–0.34	–0.34
O(egzo)	–0.36	–0.36	–0.36	–0.36	–0.36	–0.36
O(41)	–0.39	–0.39	–0.39	–0.38	–0.38	–0.39
O(61)	–0.39	–0.39	–0.39	–0.39	–0.39	–0.39
H(41)	0.21	0.21	0.21	0.21	0.21	0.21
H(61)	0.21	0.21	0.21	0.21	0.21	0.21
N(31)	–0.09	–0.09	–0.09	–0.01	–0.01	–0.33
N(32)	0.01	0.01	0.01			
N(33)	–0.01	–0.01	–0.01			
H(31)				0.77	0.77	0.24

a The atom numbering corresponds to that given in Figure S28 in SI.

The hydrogen atoms of amino **ARNH**_**2**_**OMe**, **BRNH**_**2**_**OMe**, and hydroxyl groups in sugar molecules have different electronegativities (blue region) and may act as nucleophilic centers. This implies that these hydrogen atoms can participate in the formation of H-bonds with other neighboring molecules. Alternatively, the nitrogen atoms (in the azide and amine groups) and the oxygen atoms (in the hydroxyl groups and *exo*- and *endo*-cyclic ring sites) represent the most electronegative regions and may exhibit significant electrophilic activity due to the excess negative charge. The color code of these maps ranges between −0.39 [eV] (dark red) and 0.21 [eV] (**ARN**_**3**_**OMe**, **BRN**_**3**_**OMe**) or 0.77 [eV] (**ARNH**_**2**_**OMe**, **BRNH**_**2**_**OMe**) (dark blue) in the studied compounds. As illustrated in Figure 5 and Table 5, the negative regions are primarily concentrated around the oxygen atoms, with a maximum value of −0.39 [eV]. In contrast, the maximum positive region is predominantly located over the ammonium ions, which could serve as a potential site for nucleophilic attack, with a maximum value of 0.77 [eV]. These results provide information regarding the region in which the compound can create intra- and intermolecular interactions. The negative charge on the *N*-donor of the amine group (in **ARNH**_**2**_**OMe** and **BRNH**_**2**_**OMe**) is greater (red) than that on the *N*-atom of the azido group (pale red), which aligns with the observed short contacts in the crystal structure of the amine anomers **ARNH**_**2**_**OMe** and **BRNH**_**2**_**OMe**.^46^ The most electropositive hydrogen sites being H(6), H(7), H(31), H(41), and H(61). Of these, the H(31) proton (from the protonated R-NH_3_^+^ forms of **ARNH**_**2**_**OMe** and **BRNH**_**2**_**OMe**) is more electropositive than the other protons. All carbon atoms in the pyranoside ring carry a positive charge, with C-1 exhibiting the highest positive charge density due to its position between two electronegative oxygen atoms.

Analysis of the charge distribution (Table 5) also supports the correctness of the calculated intramolecular hydrogen-bonding scheme^46^ (see Figures 4 and 5 in ref (46)), as the corresponding hydrogen donor atoms all carry positive charges and are positioned near negatively charged nitrogen or oxygen atoms.

Regions of negative charges suggest potential sites for coordination with metal ions and help localization of atoms that may be creating accepting hydrogen-bonding interactions.

### 3.5 Hydrogen-Bonding Influence on Ionization Constant

To deepen our understanding of the investigated compounds, *ab initio* calculations^46,47^ of the electronic structure and total charge distribution (Table 5 and Figure S28 in the SI) were performed under three different molecular environments, representing varying solution conditions, to better comprehend their interactions with protons. While the electronic structures of these compounds are crucial for clarifying long-range van der Waals/London dispersion interactions,^57^ their total charge distribution remains of primary importance.^58^ The latter guides the molecules into their docking configuration and ensures stability, which also depends on the detailed solution environment.^59^

The charge distribution shows that the environment is crucial for 3-amine derivatives (large differences in density and obvious protonation of the amino group), whereas, in the case of the methyl 3-azido derivatives, the electron density distribution is the same.

Based on our previous results^46,47^ for the calculated free energies of a solvated system,^60^ deprotonation reactions were studied in an aqueous solution consisting of proton transfer from a solvent molecule (in an acidic medium) onto a molecule. The nitrogen atom of the 3-amino group (in **ARNH**_**2**_**OMe** and **BRNH**_**2**_**OMe**) exhibited the highest proton affinity compared to the values obtained for both hydroxyl groups. Thus, it can be postulated that the magnitude of p*K*_*a*_ determined by the experimental methods characterizes these types of equilibria. Protonation of *endo*- and *exo-*oxygen atoms is much more difficult, so we suggest that at pH < 7, nitrogen atoms undergo protonation, but these oxygen atoms do not. Moving on to titrating toward high pH values, we suggest that only hydroxyl groups can be deprotonated. However, we are not certain which groups (primary or secondary) respond more easily. Based on the experimental values of the ionization constants, it can be seen that it depends strictly on the type of the relationship.

According to the literature,^61−63^ primary 6-OH groups are deprotonating more easily (are more acidic) than secondary −OH groups. The reactivity of secondary −OH groups, however, remains ambiguous. It is generally suggested that equatorial −OH groups in six-membered ring systems are preferentially functionalized when secondary axial partners are present. Nonetheless, the relative reactivity of monosaccharide −OH groups has been shown to depend significantly on the specific reaction conditions. Krepinsky and colleagues^64−66^ suggested that strong intramolecular hydrogen bonding could inhibit hydrogen abstraction. Brewster et al.^67^ and Houdier and Pérez^68^ used *semi*-empirical calculations to evaluate the acidities of hydroxyl groups, finding a moderate correlation between increased reactivity and the acidity of the 2-OH group.

Our previous findings^46^ proved that the intramolecular network governs the relative reactivity of hydroxyl groups in such derivatives. In methyl 3-amino-2,3-dideoxydhexopyranosides **ARNH**_**2**_**OMe** and **BRNH**_**2**_**OMe**, the formation of both inter- and intramolecular hydrogen bonds are possible because of the interactions between the hydroxyl and amino groups. For d-*ribo* isomers, stronger bonds are formed when the amine group acts as a donor. Our theoretical calculations further demonstrated that, uniquely in the case of methyl 3-amino-2,3-dideoxy-α-d-*ribo*-hexopyranoside (**ARNH**_**2**_**OMe**), an intramolecular hydrogen bond can form between the nitrogen atom of the amino group and the oxygen atom of the aglycon. This occurs due to the 1,3-*syn*-axial positions of these substituents, which reduce the distance between the donor and acceptor, creating more favorable bonding conditions (see ref (46)). The consequence of the formation of intramolecular hydrogen bonding (HB) is a decrease in the basicity of the *axial*-3-amine group in **ARNH**_**2**_**OMe**, where the lone electron pair is involved in the formation of the above-mentioned HB, and thus is less involved in proton uptake. Phenomena, such as hydrogen bonding, are very important in influencing acid strength. If we consider the effect of the relative location of the donor DHB (hydrogen atom of amine group) and acceptor AHB (oxygen atoms of *axial* methoxy and *equatorial* hydroxyl groups) in the mentioned **ARNH**_**2**_**OMe** molecule, we can see that the evaluated ionization constants (Table 2) may describe the equilibrium of intramolecular proton transfer between the atoms.

### 3.6 Binding Ability Assay of 3-Nitrogen Saccharide Derivatives with *CT*-DNA

UV–vis absorption spectroscopy has been employed as a major technique to characterize the nature and strength of interactions between 3-nitrogen saccharide derivatives and *CT*-DNA. Detailed qualitative analysis of the spectral results included monitoring of shifts in the maximum position (bathochromic or hypsochromic effect) and changes in its absorbance (hyperchromism or hypochromism) when the saccharide derivatives are in free form in solution to when compounds are bound to nucleic acids. The magnitude of these changes is associated with the strength of the sugar—DNA interaction.

**Figure 6:**
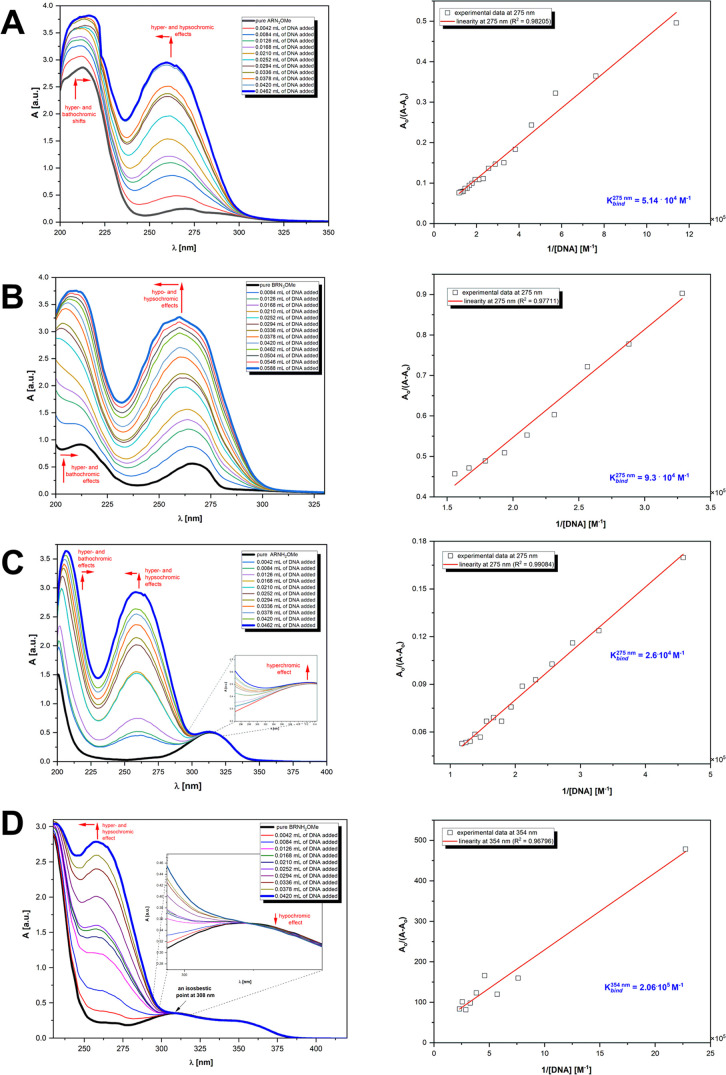
Absorption spectra of compounds studied in the presence of increasing amounts of *CT*-DNA: **A**. ARN_3_OMe; **B**. BRN_3_OMe; **C**. ARNH_2_OMe; **D**. BRNH_2_OMe. Arrows indicate absorbance changes upon increasing *CT*-DNA concentration. Right: plot of *A*_o_/(*A* – *A*_o_) = *f*(1/[DNA]) established as a result of both compound interactions through titration with *CT*-DNA in Tris-HCl buffer (5 mM/50 mM NaCl, pH 7.43).

Spectrophotometric titrations of 3-nitrogen saccharide derivatives were recorded in 5.00 mM TRIS-HCl buffer pH 7.32 at room temperature in both the absence and presence of varying *CT*-DNA concentrations (Figure 6).

As shown in Figure 6A, the free form of the methyl 3-azido-2,3-dideoxy-α-D*-ribo*-hexopyranoside (**ARN**_**3**_**OMe**) displayed absorption bands at 200–400 nm with two maxima at λ_1_ = 213 nm and λ_2_ = 270 nm. The chromophoric groups of adenine, guanine, cytosine, and thymine exhibited a broad band in the range of 200–350 nm in the spectrum of *CT*-DNA with maximum absorption at 260 nm as a result of *n →* π* and π → π* transitions. Upon increasing the nucleic acid concentration in 6.44 mM **ARN**_**3**_**OMe** solution, hypsochromic shifts of the distinguished bands in the direction of lower wavelengths (−9 nm < Δλ < −3 nm) were observed along with an increase in absorbance. Further addition of *CT-*DNA resulted in a change in band position in the far-UV region. A bathochromic shift of λ_1_ from 210 to 215 nm (Δλ = +5 nm) was observed with a simultaneous increase in its intensity. The observed during titration bathochromic and hypsochromic shifts, accompanied by hyperchromicity, likely indicate an intercalation binding mode between the methyl 3-azido-α-d-*ribo* saccharide derivative and DNA base pairs, by reducing the π → π* transition energy.^69^ Additionally, the appearance of a hyperchromic effect in the spectra of titled compounds during saccharide—DNA adduct formation indicates that electrostatic interactions are feasible.^70^ This hyperchromic effect may also result from the disruption of hydrogen bonds that stabilize the DNA double helix, thereby diminishing the resonance of the aromatic rings.^71^

An increase in band intensity could also arise from distortion in DNA conformation induced by methyl 3-nitrogen saccharide derivative adduct formation, which exposes the purine and pyrimidine nucleobases and causes higher absorbance values.^72^

The change in the configuration of the anomeric carbon resulted in a slightly different spectral effect, as described below. During the addition of a small amount of *CT-*DNA to the free **BRN**_**3**_**OMe** solution, the position and intensity of the absorption bands changed as follows. The unbound methyl 3-azido-β-d-*ribo* saccharide derivative exhibited two maximum absorptions, corresponding to λ_1_ = 212 nm and λ_2_ = 267 nm, respectively (Figure 6B). Upon increasing the concentration of *CT*-DNA in the system studied, the absorption band at 212 nm flattened. Further addition of a small amount of nucleic acid resulted in the formation of a new maximum at 203 nm, which was shifted in the direction of higher wavelengths (Δλ = +5 nm) along with the hyperchromic effect of absorbance. Moreover, it was found that the adsorption maximum at λ_2_ = 267 nm during spectrophotometric titration underwent a blue shift to 261 nm (Δλ = −6 nm) with simultaneous hyperchromism of intensity. Hyperchromism may suggest the occurrence of electrostatic interactions between the positively charged saccharide derivative and the negatively charged phosphate backbone along the periphery of the *CT*-DNA double helix.^73^ The replacement of the azido substituent with the amine group at the third position of the saccharide skeleton caused similar spectral alterations during titration of the 3-nitrogen saccharide derivative with *CT*-DNA, with one distinguishing difference. The free form of **ARNH**_**2**_**OMe** exhibited two sharp absorption bands at λ_1_ = 201 nm and λ_2_ = 314 nm (Figure 6C).

As in the case of the azido saccharide derivative, the bathochromic shift in the direction of higher wavelengths from 201 to 210 nm (Δλ = +9 nm) with a gradual increase in absorbance (hyperchromism) was recorded. Increasing the concentration of *CT*-DNA in **the ARNH**_**2**_**OMe system** resulted in the appearance of a new band at 261 nm, which underwent a blue shift toward shorter wavelengths with a simultaneous increase in its intensity. In contrast to the methyl 3-azido saccharide derivatives, in the spectrum of **ARNH**_**2**_**OMe** no change in the band position or magnitude of absorbance at 314 nm was registered during the stepwise addition of *CT-*DNA in the system studied. Many of the hydrogen-bonding sites of DNA are accessible in the major and minor grooves of the double helix.

The amine group at the third position of the saccharide skeleton can form a hydrogen bond with the N(3) nitrogen of adenine and O(2) oxygen of thymine, which can be assigned to the hyperchromic effect observed during titration.^74^

Groove binders possess a higher specificity for AT-rich regions. This is probably due to the stronger van der Waals interactions between the saccharide derivatives and groove walls in this electronegative AT pocket. Moreover, this common specificity to the adenine-thymine groove region is because it is narrower than the guanine-cytosine region, which is sterically hindered by the amino group at C(2) of the guanine base.^44^

The change in the configuration of the anomeric carbon and the presence of an amine moiety at the third position of the saccharide ring resulted in additional spectral effects. The unbound form of the **BRNH**_**2**_**OMe** derivative possessed three absorption bands, one in the far-UV region at λ_1_ = 222 nm and two in the near-UV region at λ_2_ = 314 nm and λ_3_ = 350 nm (Figure 6D). When increasing the amount *of CT*-DNA introduced into the 3-amino-2,3-dideoxy-β-d-*ribo*-hexopyranoside solution, new bands appeared at 262 nm and underwent blue shifts (hypsochromic effect) toward lower wavelengths (Δλ = −4 nm) with a clear increase in absorbance. As described earlier, hyperchromism could be assigned to damage of the double helix, which allows the 3-amino-β-d-*ribo* derivative to be embedded between base pairs. Consequently, changes in base-stacking forces were observed.^75^ In response to the increase in π-orbital electron density and the energy gap between π and π* orbitals, a hypsochromic effect coupled with hyperchromism was observed in the spectrum of **BRNH**_**2**_**OMe** responsible for the electrostatic interaction in the system studied.^76^ This observation is consistent with the previously described spectral changes of the examined **ARNH**_**2**_**OMe**. It should be emphasized that different alterations were observed in the near-UV region of the **BRNH**_**2**_**OMe** spectrum. First, in the case of the maximum absorption at 350 nm, the hypochromic effect of intensity was recognized with no apparent wavelength shift. Usually, the decrease in absorbance is due to conformational changes in the *CT*-DNA helix axis caused by surface binding between saccharide derivatives and nucleic acids.^77^ Second, the stepwise addition of *CT*-DNA to free **BRNH**_**2**_**OMe** resulted in the appearance of an isosbestic point at 308 nm, indicating the establishment of an equilibrium between the sugar derivative and the nucleic acid.

As described above, the spectral alteration after the stepwise addition of DNA to the methyl 3-nitrogen saccharide derivative solution is considered to occur with changes in the structure and conformation of the DNA double helix. When the interaction between the titled compounds and nucleic acids is favorable, a hypochromic effect (decrease in DNA absorption) is observed. Electrostatic interactions increase in DNA intensity (hyperchromism). The hyperchromic effect may also arise from the disruption of hydrogen bonds that stabilize the double-helix structure of DNA. Qualitative analysis showed that 3-nitrogen saccharide derivatives are major groove DNA binders, which present a specific preference for the ATAT-rich region. The obtained results showed that the configuration of the asymmetric centers in the sugar skeleton may also influence the orientation of the saccharide derivatives and their exposure toward the minor groove.

The spectral data obtained from electronic absorption titrations allowed us to determine the ability and strength of interactions of the methyl 3-nitrogen saccharide derivatives with *Calf Thymus* DNA. The plot of *A*_o_/(*A* – *A*_o_) versus 1/[DNA] yielded a linear relationship, and the binding constant value was determined from the ratio of the intercept to the slope using these linearity parameters (Figure 6).

The determined binding constant values (Table 6) for all 3-nitrogen saccharide derivatives were in the range 2.60 × 10^4^ M^–1^–2.06 × 10^5^ M^–1^. Based on the obtained data, the title compounds were arranged in order according to their decreased interaction strength with *CT*-DNA as follows: **BRNH**_**2**_**OMe** > **BRN**_**3**_**OMe** > **ARN**_**3**_**OMe** > **ARNH**_**2**_**OMe**. The *K*_bind_ values determined for all examined compounds correlated well with the changes observed in the UV–vis spectra, suggesting that the title compounds likely interacted with DNA through groove binding and partial intercalation, supplemented by electrostatic interactions. Among the binding results, **BRNH**_**2**_**OMe** possessed the strongest binding potency with DNA base pairs (*K*_bind_ = 2.06 × 10^5^ M^–1^), while the 3-amine-α-d-*ribo* saccharide derivative (*K*_bind_ = 2.60 × 10^4^ M^–1^) exhibited the lowest strength of interaction with biomolecule studied.

**Table 6:** Spectral characteristics of 3-nitrogen saccharide derivatives bound to *CT*-DNA with intrinsic binding constant values K_*bind*;_ the footnotes

	λ_max_ [nm]			*K*_bind_ [M^–1^]
compound	free	bound	Δλ	BH method
**ARN**_**3**_**OMe**	270	261	– 9	5.14 × 10^4^
	213	210	+ 3	
**BRN**_**3**_**OMe**	267	261	+ 6	9.30 × 10^4^
	212	208	– 4	
**ARNH**_**2**_**OMe**	261^a^	259	– 3	2.60 × 10^4^
	201	210	+ 9	
**BRNH**_**2**_**OMe**	262^a^	258	– 4	2.06 × 10^5^
	308	311	+ 3	

a Denote a new band.

This observation strongly implies that the *CT*-DNA-binding affinity and/or specificity varies between the α-d-*ribo* and β-d-*ribo* derivatives.

Therefore, subtle differences in the sugar residue structure may have implications for *the CT*-DNA-binding mode. Careful quantitative analysis indicated that β-linkages in the saccharide moiety may be structurally required for DNA groove recognition in aqueous media.^78^ The obtained results suggest that the positively charged methyl 3-azido-2,3-dideoxy-α-d-*ribo* saccharide derivatives groove binder interacts with AT-rich regions 1.98-fold more effectively than neutral 3-amine sugar derivatives. The cationic nature of the 3-azido saccharide analogs could promote a better fit in the DNA groove and enhance the stability of the double helix. It should be noted that potential groove blockage and/or induction of conformational changes prevents transcription factor binding.^79^ In the case of the β configuration of the anomeric carbon, the saccharide derivative with the amine group at the third position exhibited a 2.22-fold stronger affinity to *CT*-DNA than its azido analog. The 3-NH_2_ group of the sugar ring can form hydrogen bonds with N(3) of adenine and O(2) of thymine within the groove regions. Additionally, the amine group in the saccharide residue contributes to a well-structured major groove hydration network, involving van der Waals forces and several strong hydrogen bonds to the DNA double helix.^80,81^ The binding mode of carbohydrates is not restricted to specific nucleobases but generally favors the 5′ side of purines and the 3′ side of pyrimidines.^82,83^

### 3.7 Molecular Docking Results

**Figure 7:**
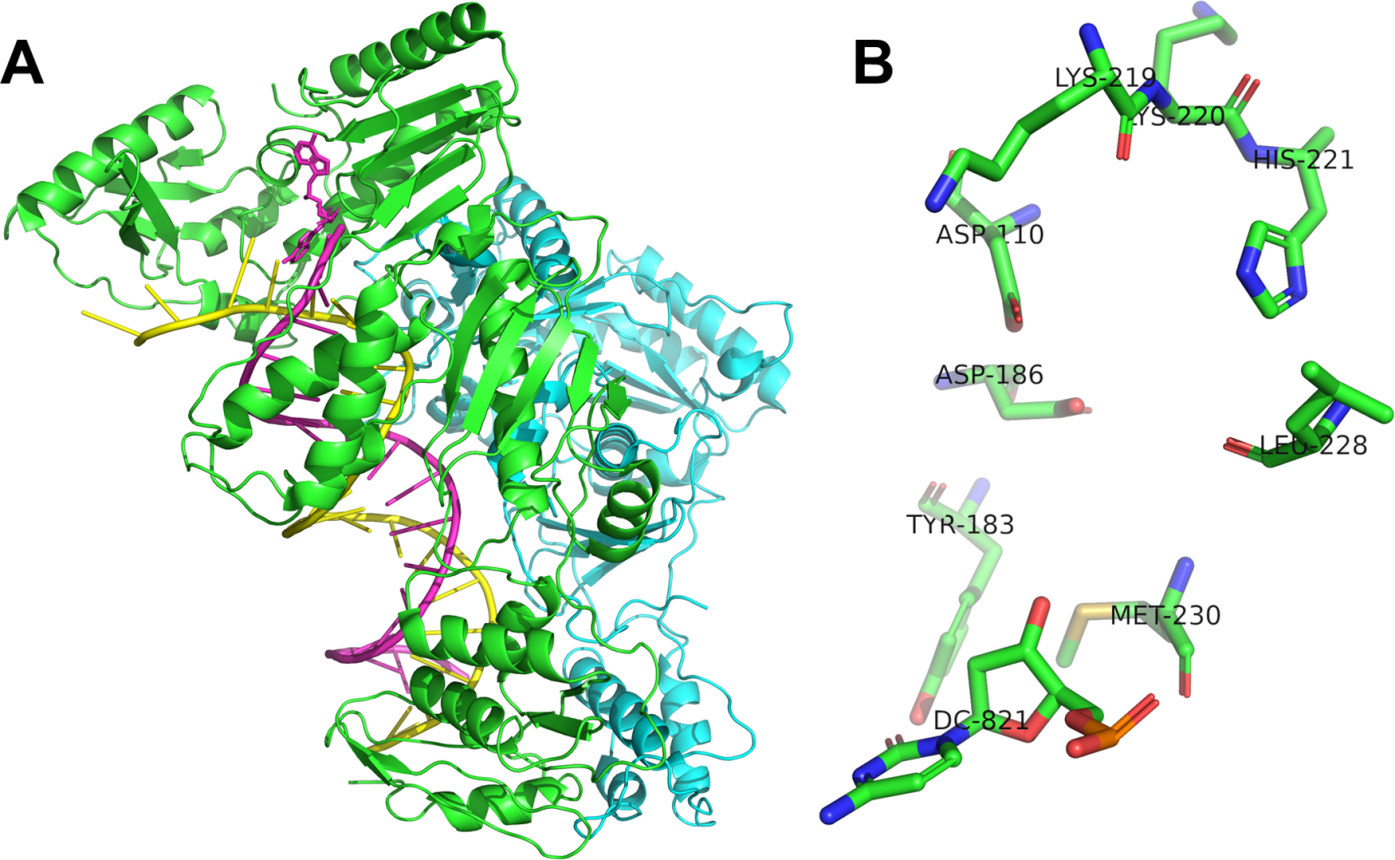
**A**. Schematic representation of RT HIV tertiary structure (PDB code 1T03 without antibodies) and **B**. its active site.

The derivatives of 2,3-dideoxy-d-*ribo*-hexopyranosides with methoxyl groups as aglycones (*O*-glycosides) were tested for their biological properties. These compounds have been proposed to interact with the active region of the HIV enzyme reverse transcriptase (RT) to inhibit virus replication in infected cells. According to this prediction, the use of these compounds would result in AZT-like inhibition of HIV reverse transcriptase. The four compounds docked to HIV-1 RT were the methyl 3-azide derivatives (**ARN**_**3**_**OMe** and **BRN**_**3**_**OMe**) and their 3-amine derivatives (**ARNH**_**2**_**OMe** and **BRNH**_**2**_**OMe**). RT HIV-1 structure is shown in Figure 7.

There were two purposes for the molecular docking experiment: *in silico* confirmation of the possibility of direct interaction of the tested AZT analogs with the active center of the RT enzyme, and quantitative estimation of the free enthalpy Δ*G* of the interaction of each analog with the RT active site. Negative values of free enthalpy indicate the possibility of spontaneous formation of such complexes in nature. In our research, we showed that the compounds described earlier interact with the active center of HIV-1 reverse transcriptase and, while blocking this active center, they compete with standard nucleotides. Each of the tested AZT analogs showed a characteristic interaction profile precisely in the RT active center. Based on the similarity of this compound to AZT and their possibility for interactions within the RT active site, we indicate that their biological properties allow their binding to nucleic acids via covalent bonds, blocking their expansion and inhibiting HIV-1 reverse transcriptase, and, as a consequence, making it difficult or impossible to form new virus particles. Each of the tested analogs had a potential phosphorylation site, which determined its incorporation into the nucleic acid structure.

**Figure 8:**
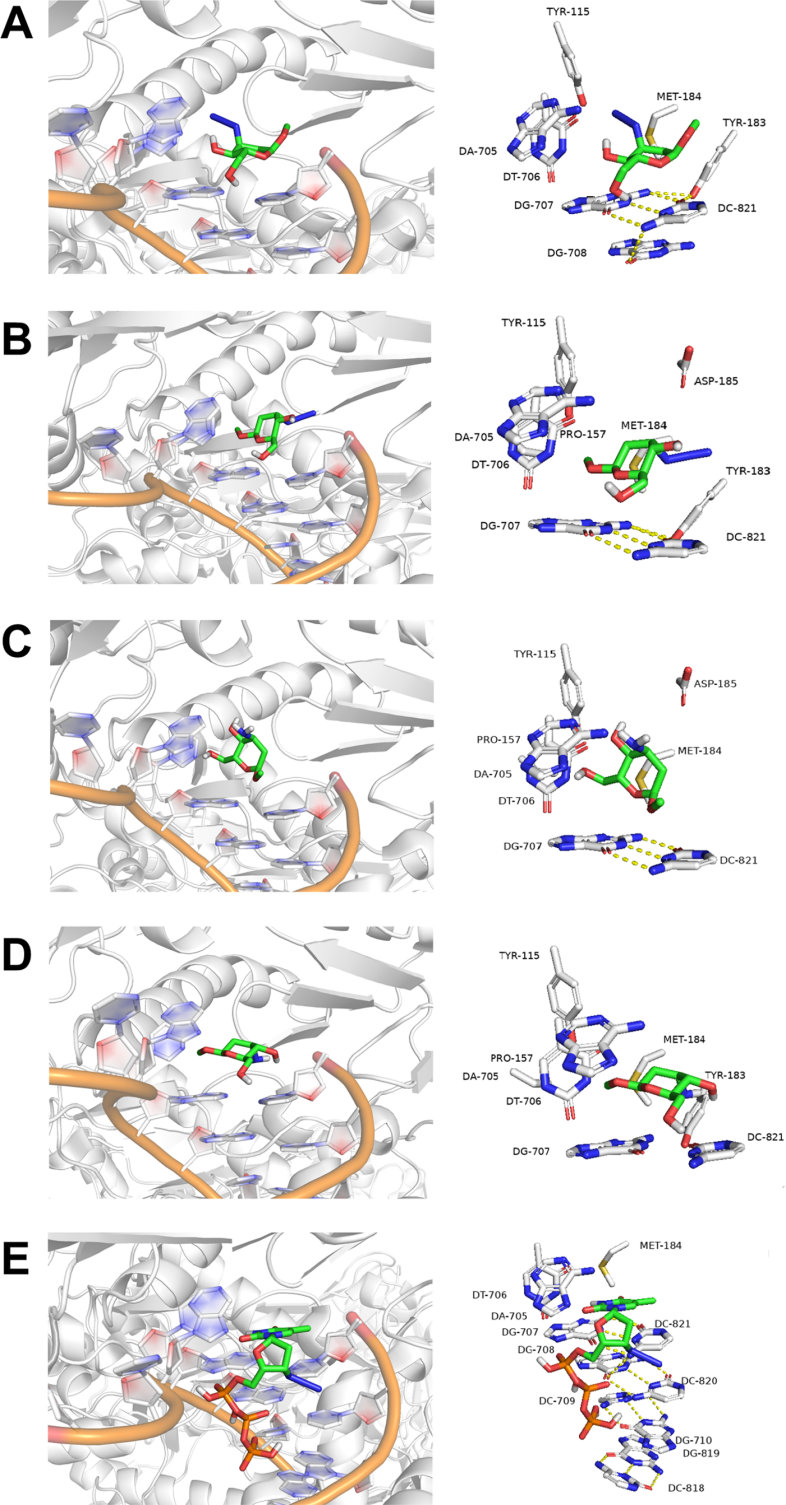
Illustration of the compound position of the enzyme active site for **A**. ARN_3_OMe, **B**. BRN_3_OMe, and **C**. ARNH_2_OMe, **D**. BRNH_2_OMe, and **E**. AZT.

In our research, we performed molecular docking–multiple random incorporation of the docked molecule into RT– and identified conformations with the lowest free enthalpy Δ*G*.^1^ Because of the applied docking method (a genetic algorithm using Lamarck theory), each subsequent complex configuration was better optimized and more reminiscent of actual complexes occurring in nature. Biopolymer structures did not change; therefore, the ligands could change their conformation to a limited extent during docking. For the ligands, ring formation was preserved in each case. During docking, the position and orientation of the ligand relative to the immobile biopolymer were changed. Models of these compounds were docked in molecular docking simulations to the crystallographic structure of RT HIV-1, as shown in Figure 8.

The following description includes the molecular docking results for two synthesized pairs of 2,3-dideoxy-d-*ribo*-hexopyranosides anomers, described as **ARNH**_**2**_**OMe, BRH**_**2**_**OMe**, and their azide precursors **ARN**_**3**_**OMe** and **BRN**_**3**_**OMe**, which are alternatively called AZT analogs.

The results are summarized in Table 7.

**Table 7:** Nucleotides and Amino Acid Residues are Directly Involved in Interactions with *O*-Methyl Glycosides

**ARN**_**3**_**OMe**	**ARNH**_**2**_**OMe**	**BRN**_**3**_**OMe**	**BRNH**_**2**_**OMe**	**AZT**
Δ*G* = −6.0 kcal/mol	Δ*G* = −6.2 kcal/mol	Δ*G* = −6.0 kcal/mol	Δ*G* = −5.8 kcal/mol	Δ*G* = −10.0 kcal/mol
**DNA**				
DA T 705	DA T 705	DA T 705	DA T 705	DA T 705
DT T 706	DT T 706	DT T 706	DT T 706	DT T 706
DG T 707	DG T 707	DG T 707	DG T 707	DG T 707
DG T 708	-	-	-	DG T 708
-	-	-	-	DC T 709
-	-	-	-	DG T 710
-	-	-	-	DC P 818
-	-	-	-	DG P 819
-	-	-	-	DC P 820
DC P 821	DC P 821	DC P 821	DC P 821	DC P 821
**PROTEIN**				
TYR A 115	TYR A 115	TYR A 115	TYR A 115	-
-	PRO A 157	PRO A 157	PRO A 157	-
TYR A 183	-	TYR A 183	TYR A 183	-
MET A 184	MET A 184	MET A 184	MET A 184	MET A 184
-	ASP A 185	ASP A 185	-	-

Table 7 shows amino acid residues from the active site of HIV reverse transcriptase, which is within a distance of 5 Å from the docked deoxysaccharide derivative with the *O*-methyl group as an aglycone. The letter and number designations used and applied constitute an accepted arbitrary convention (based on a file with protein structure with PDB code 1T03(84)). Table 7 includes the following: interaction-free enthalpy Δ*G* for complex configurations with the lowest energy, information about amino acid residues or nucleotides, name of peptide or nucleic chain, and the number of amino acid residues or nucleotides. The last column contains a list of amino acid residues and nucleotides within a distance of 5 Å from the AZT molecule. It is worth noting the slightly different docking method of AZT compared to that of other compounds from this group. These differences arise from the presence of a relatively large group (thymine) in the AZT molecule. Some differences were also observed in a group of four deoxysaccharide derivatives with an *O*-methyl group as an aglycone. The second compound (**BRN**_**3**_**OMe**) interacted with the largest number of amino acid residues in the protein active site, whereas the smallest number of these interactions was observed for the first compound (**ARN**_**3**_**OMe**). The other two compounds behaved similarly (except for individual amino acid residues). All low-energy complexes of deoxysaccharide derivatives (and AZT) were characterized by negative values of free enthalpy of the interactions of these compounds with HIV reverse transcriptase, which provides grounds for the spontaneous formation of complexes and confirms the use of these synthesized *O*-glycosides as inhibitors for the tested enzyme.

## 4 Conclusions

The presence of heteroatoms such as nitrogen in sugar molecules enables the implementation of pH-spectrophotometric and potentiometric microtitration methods to evaluate protolytic equilibria and determine p*K* values. Our experimental investigation confirmed our previously published theoretical calculations^37,46,47^ and strictly indicated that the methyl 2,3-dideoxy-3-nitrogen sugar derivatives studied may exist in more than one form in aqueous solutions. Analysis of the relative activities of both hydroxyl groups and 3-amino-α-D-anomer **ARNH**_**2**_**OMe** suggested that intramolecular hydrogen-bonding networks, involving the carbohydrate DHB (amine group as donor–acceptor) and AHB (oxygen as a hydrogen acceptor), play a crucial role. Based on these insights, we demonstrated that the delocalization of positive charge through the hydrogen-bonding network is a key factor in determining the relative activities of carbohydrates hydroxyl groups. The MARVIN software was utilized to predict the total charge distributions of the molecules studied. The resulting atomic charge distribution was used to qualitatively interpret electrophilic or nucleophilic attack, H-bond interactions, and to define regions of local negative and positive potential in the molecules. Understanding these charge distributions provides insight into how molecules interact with one another.

Based on our previous theoretical and experimental studies, we hypothesized that the p*K* determination methods employed in this work would yield reliable p*K* values for the methyl 3-nitrogen sugar derivatives under investigation, thereby validating the calculated protonation and deprotonation centers. Generally, in a basic medium, the secondary hydroxyl groups of such molecules undergo deprotonation, whereas in an acidic medium, the nitrogen atom is protonated.

In the performed experiment We also determined the stability constants for the title compounds with Cu(II) and Ni(II) ions in a water solution. Our results show azido saccharides are more likely to create complexes with Cu(II) ion, while aminosaccharides with Ni(II). The proposed equilibrium models explain the possibility of saccharides binding to metal ions. The most likely chelating sites in the ligands were the azido (3-N_3_), amine (3-NH_2_), and hydroxyl (4-OH) groups. Stoichiometric models of the formed complexes were proposed based on *A*-diagrams and titration results.

The affinity of the methyl 3-nitrogen saccharide derivatives to *Calf Thymus* DNA was evaluated qualitatively by electronic absorption titration based on chromic effects analyses. The obtained results suggest that DNA-binding ability and/or specificity are dependent on the conformation of the chiral centers and type of substituent at the third position of the saccharide skeleton. Subtle changes in the sugar structure may have influenced the strength of the nucleic acid-binding mode. On the other, the molecular docking results and findings show effective binding to the RT active site supports the potential use of these synthesized *O*-glycosides as inhibitors of HIV reverse transcriptase. While all tested compounds show potential as inhibitors, variations in interaction profiles suggest differing levels of efficacy. The compound **BRN_3_OMe**, in particular, shows the highest interaction with enzyme active site residues and also with DNA indicating it may be the most effective among the derivatives tested.

By integrating experimental and computational methods, we uncover novel sugar-based inhibitors of HIV-1 reverse transcriptase, contributing to the ongoing efforts to develop new antiretroviral therapies. The findings from these studies could pave the way for further experimental validation and optimization of these compounds, ultimately leading to more effective treatments for HIV-1 infection.

## Supporting Information Available

IR (Figures S1–S4) and NMR spectra for structure confirmation (Figures S5–S12); Figures S13 and S14 show the solution spectra of compounds studied at the selected pH; Figures S15 and S16 present selected *A*-diagrams (**A**) and a plot of absorbance at selected wavelengths vs. pH (**B**) for compounds studied; Figures S17–S21 show spectrophotometric and potentiometric titration curves and the corresponding microspecies distribution (%) diagram at the given pH for the compounds studied, respectively; Figure S22 shows the absorption spectrum of M(II) in an aqueous solution upon complexation with the compounds studied; Figures S23–S26 presents the results of the complexation process between compounds studied and Cu(II) or Ni(II) ions; Figure S27 shows selected *A*-diagrams for the complexes with saccharides; Figure S28 shows the atom numbering of the studied compounds (PDF)
