# Supplementary material for: Comprehensive Chemical Analysis of the Methyl 3-Nitrogen-2,3-Dideoxysaccharides Derivatives with d-ribo-Configuration: Synthesis, Reactivity of HIV-1 Reverse Transcriptase Inhibitors
Source: J Phys Chem B. 2025 Jan 14;129(3):911–29. doi: 10.1021/acs.jpcb.4c08136 (PMC11770755; doi:10.1021/acs.jpcb.4c08136)
Supplement: Supplementary file 1 — jp4c08136_si_001.pdf [file jp4c08136_si_001.pdf]

Supporting Information

Comprehensive Chemical Analysis of the Methyl 3-Nitrogen-2,3-Dideoxysaccharides Derivatives with D-*ribo*-configuration: Synthesis, Reactivity of HIV-1 Reverse Transcriptase Inhibitors

Aleksandra M. Dąbrowska[1,*], Rajmund Kaźmierkiewicz[2], Anna M. Barabaś-Lepak[3], Małgorzata Biedulska[4], Agnieszka Chylewska[1,*]

[1] *Intermolecular Interaction Laboratory, Department of Bioinorganic Chemistry, Faculty of Chemistry, University of Gdańsk, Wita Stwosza 63, 80-308 Gdańsk, Poland*
[2] *Laboratory of Biomolecular Systems Simulations, Intercollegiate Faculty of Biotechnology, University of Gdańsk and Medical University of Gdańsk, Abrahama 58, 80-307 Gdańsk, Poland*
[3] *I Secondary School named after Maria Skłodowska-Curie in Tczew, Maritime School 1, 83-110 Tczew*
[4] *Institute of Biotechnology and Molecular Medicine, Trzy Lipy 3, 80-172 Gdańsk, Poland*

*Correspondence: aleksandra.dabrowska@ug.edu.pl, agnieszka.chylewska@ug.edu.pl

The Supporting Information *SI* (pdf file) contains an additional 28 Figures.

■ AUTHORS INFORMATION

Aleksandra M. Dąbrowska
orcid.org/0000-0002-1953-6636;
E-mail: aleksandra.dabrowska@ug.edu.pl

Rajmund Kaźmierkiewicz
orcid.org/0000-0002-7066-3518;
E-mail: rajmund.kazmierkiewicz@biotech.ug.edu.pl

Anna M. Barabaś-Lepak
orcid.org/0000-0001-6160-6813;
E-mail: anna.barabas.lepak@1lotczew.pl

Małgorzata Biedulska
orcid.org/0000-0002-0659-7659
E-mail: m.biedulska@ibmm.pl

Agnieszka Chylewska
orcid.org/0000-0001-7413-1503;
E-mail: agnieszka.chylewska@ug.edu.pl

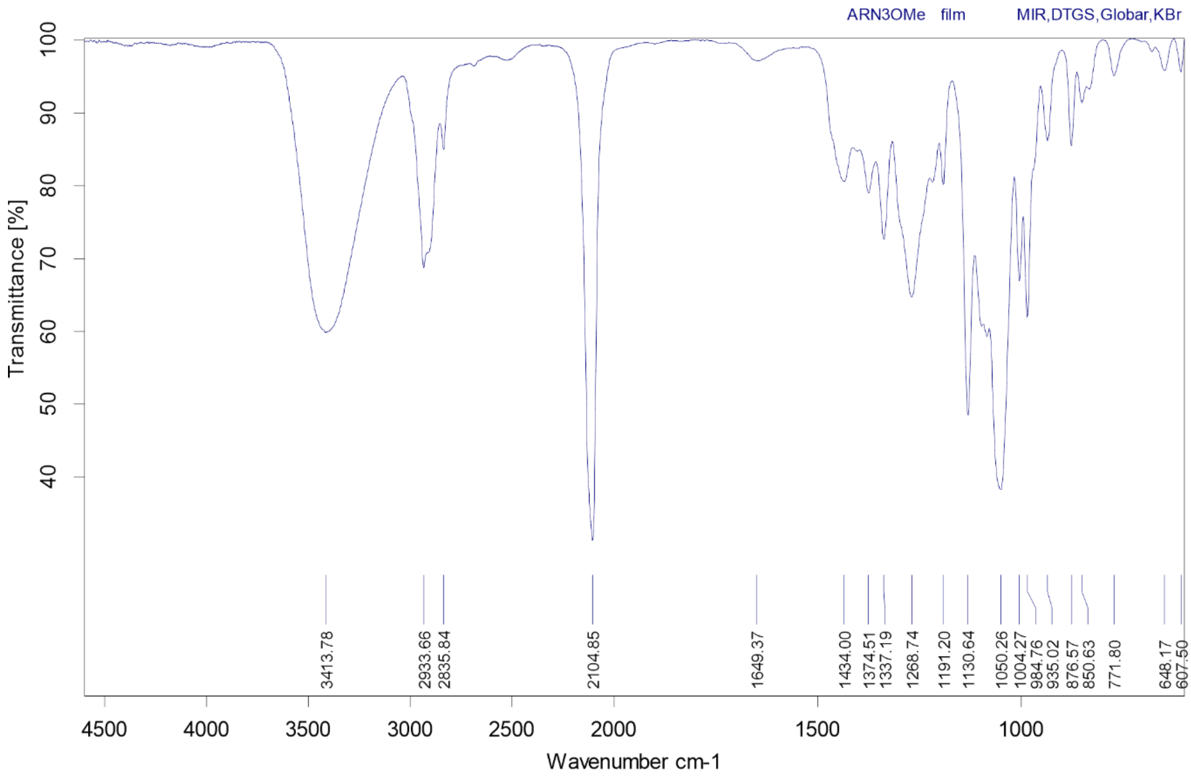

Figure S1. IR spectrum of the compound **ARN$_3$OMe** registered in film (KBr).

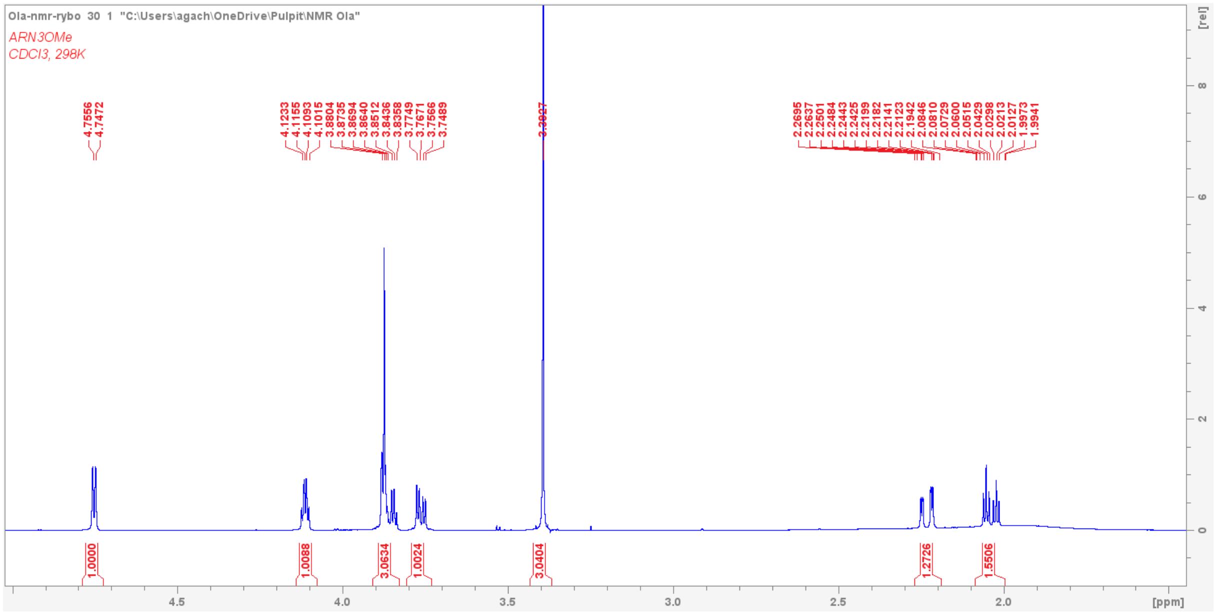

Figure S2. ^{1}H NMR spectrum of the compound **ARN$_3$OMe** registered in $CDCl_3$; 298 K.

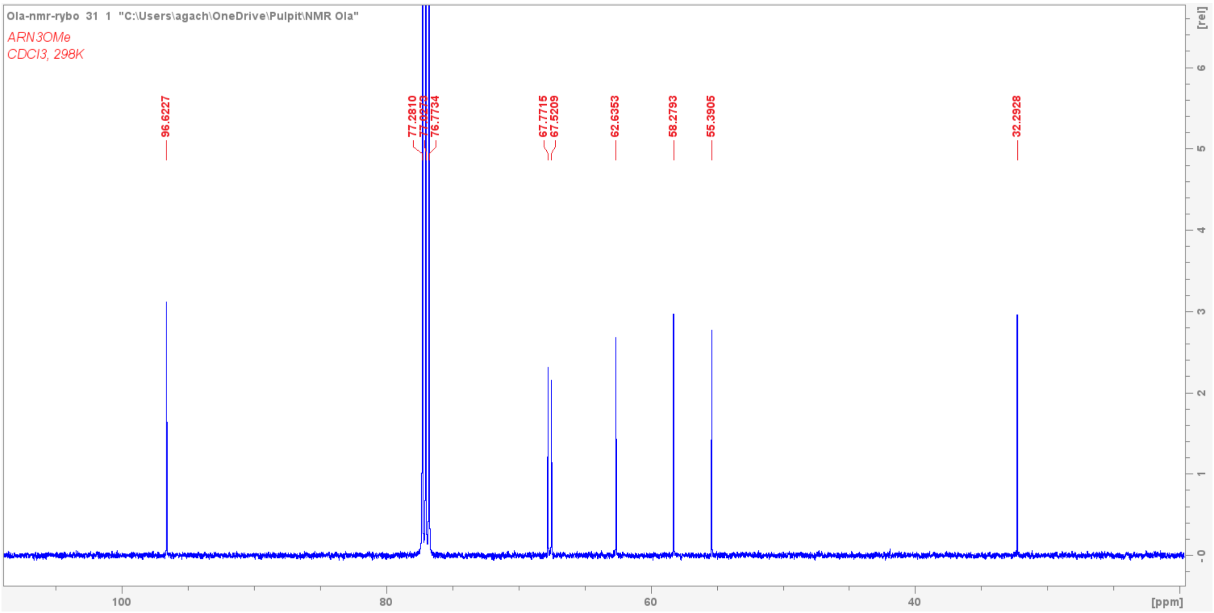

Figure S3. ^{13}C NMR spectrum of the compound **ARN₃OMe** registered in $CDCl_3$; 298 K.

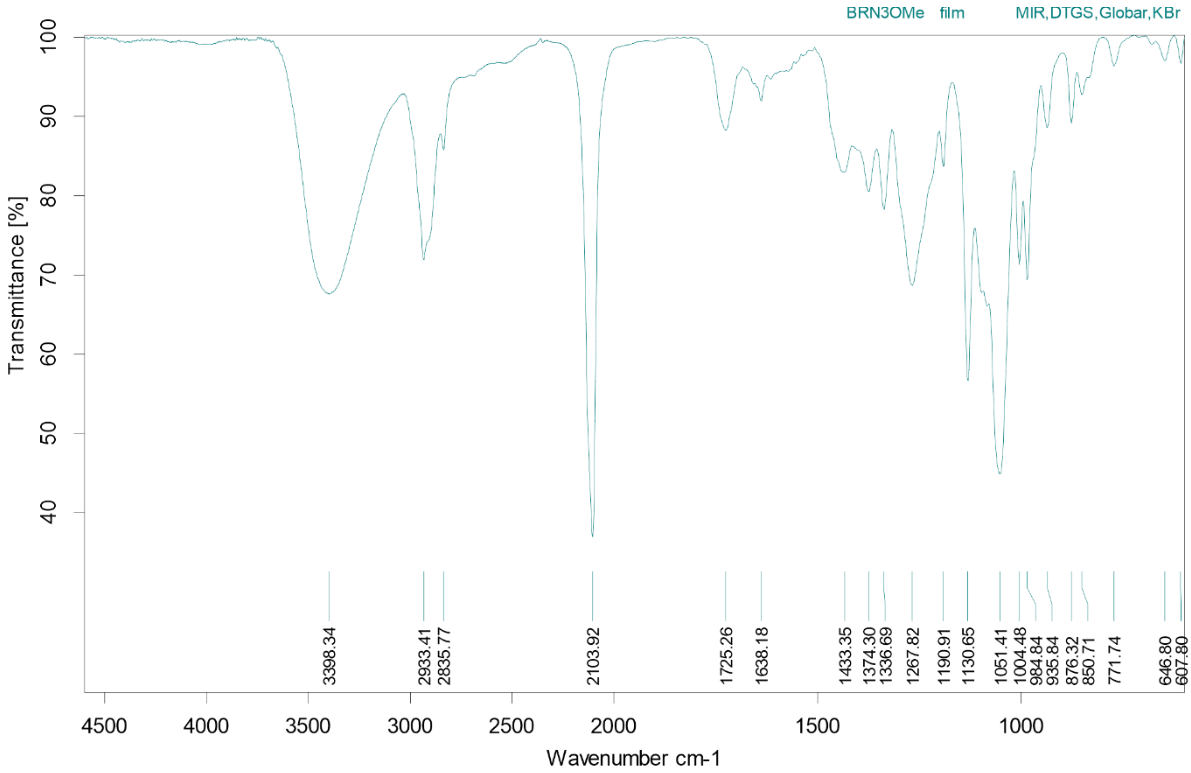

Figure S4. IR spectrum of the compound **BRN₃OMe** registered in film (KBr).

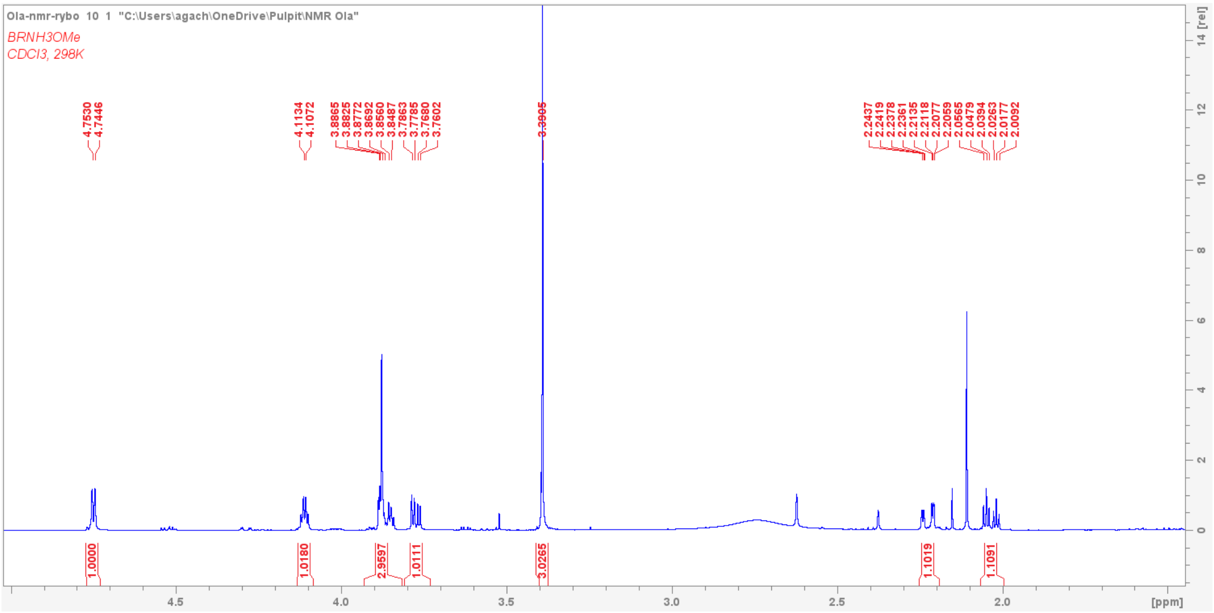

Figure S5. ^{1}H NMR spectrum the compound **BRN$_3$OMe** registered in $CDCl_3$; 298 K.

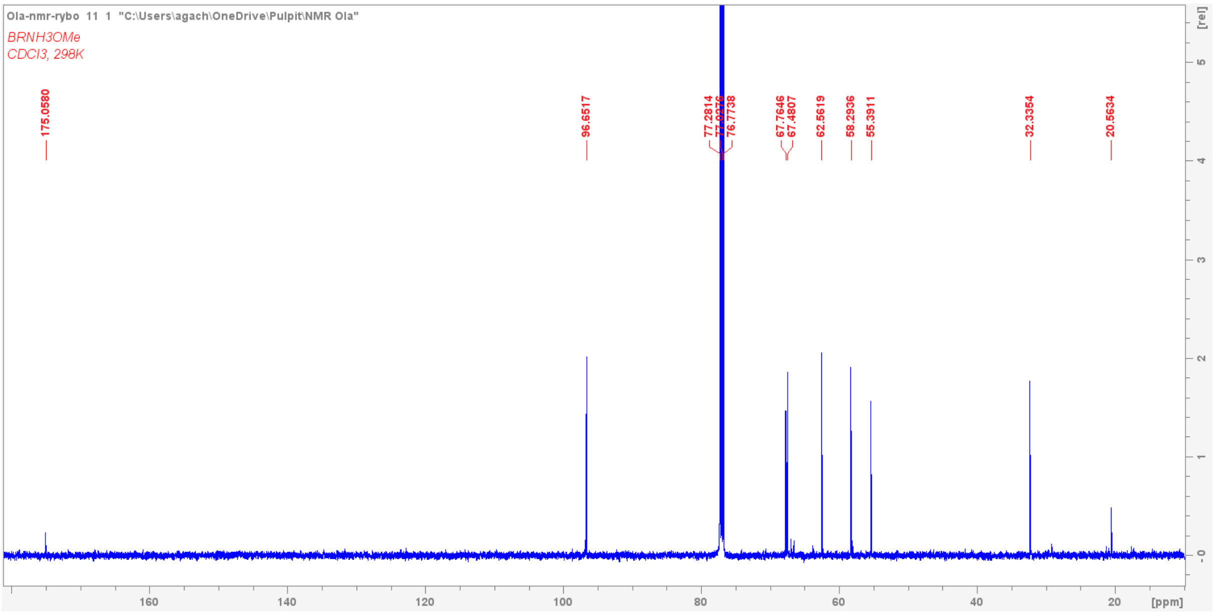

Figure S6. ^{13}C NMR spectrum of the compound **BRN$_3$OMe** registered in $CDCl_3$; 298 K.

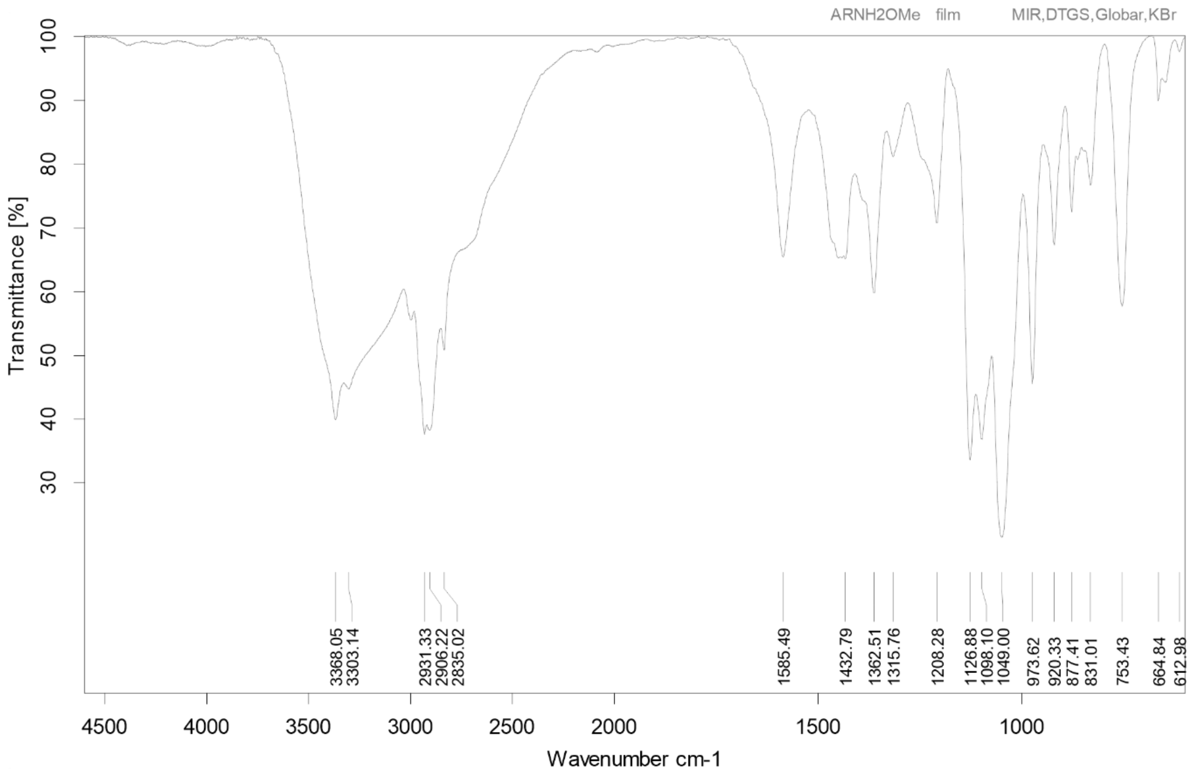

Figure S7. IR spectrum of the compound **ARNH$_2$OMe** registered in film (KBr).

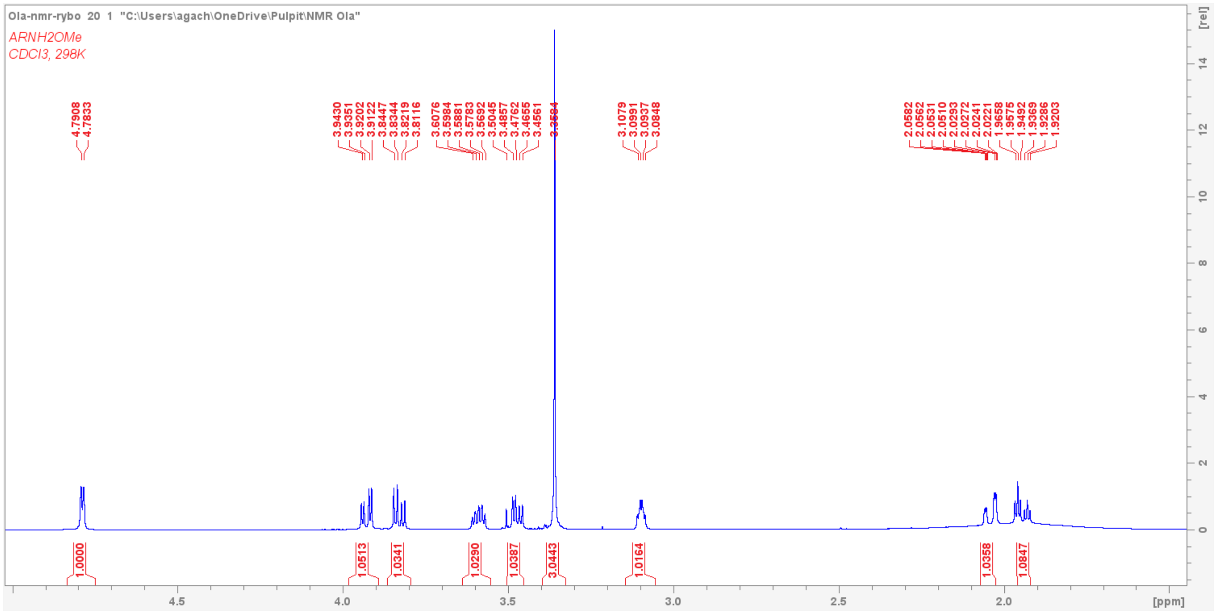

Figure S8. ^{1}H NMR spectrum of the compound **ARNH$_2$OMe** registered in $CDCl_3$; 298 K.

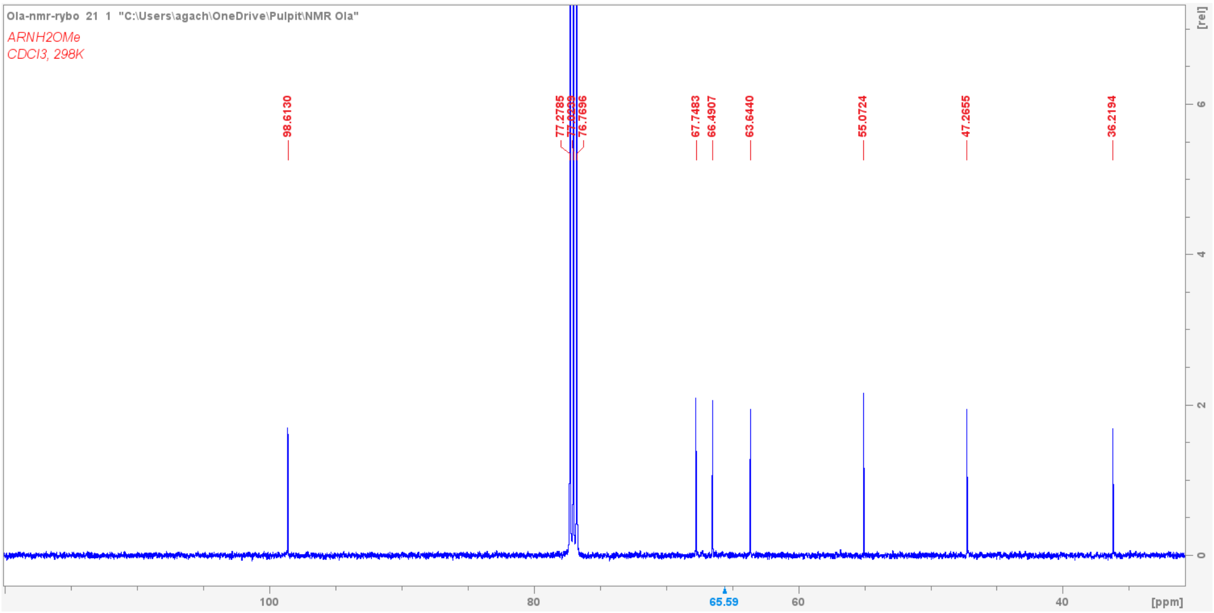

Figure S9. ^{13}C NMR spectrum of the compound **ARNH$_2$OMe** registered in $CDCl_3$; 298 K.

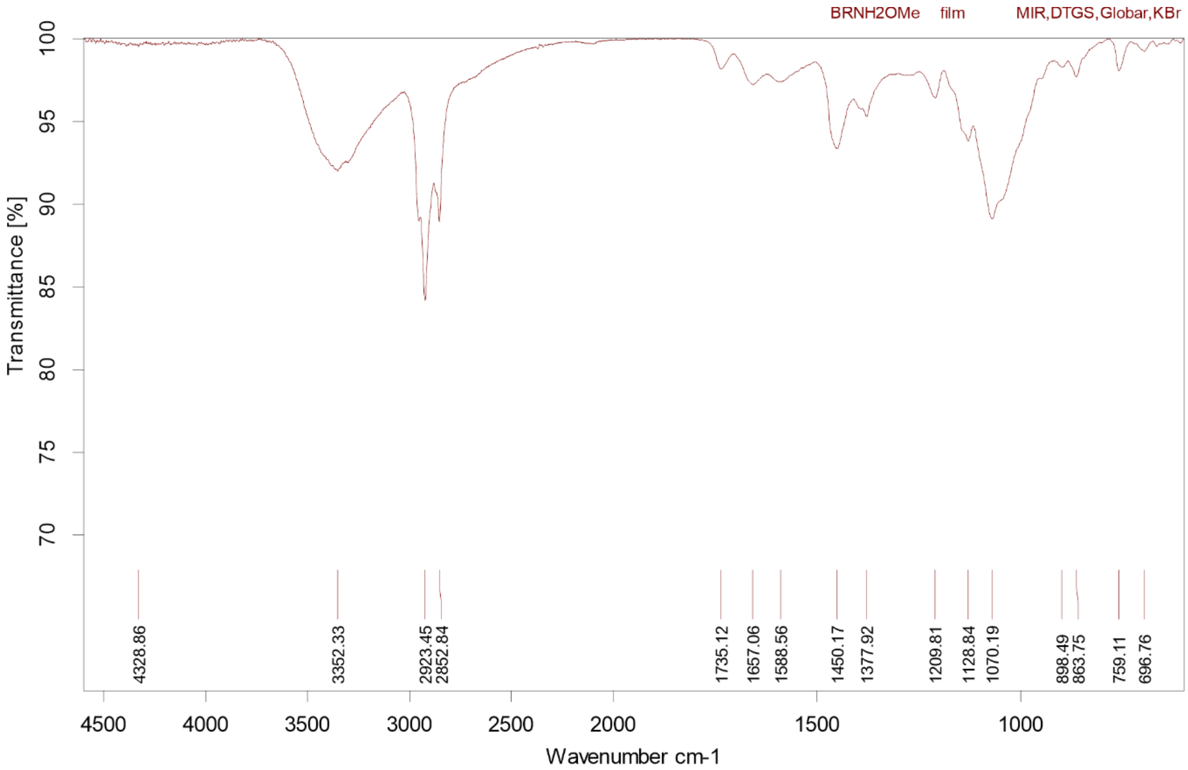

Figure S10. IR spectrum of the compound **BRNH$_2$OMe** registered in film (KBr).

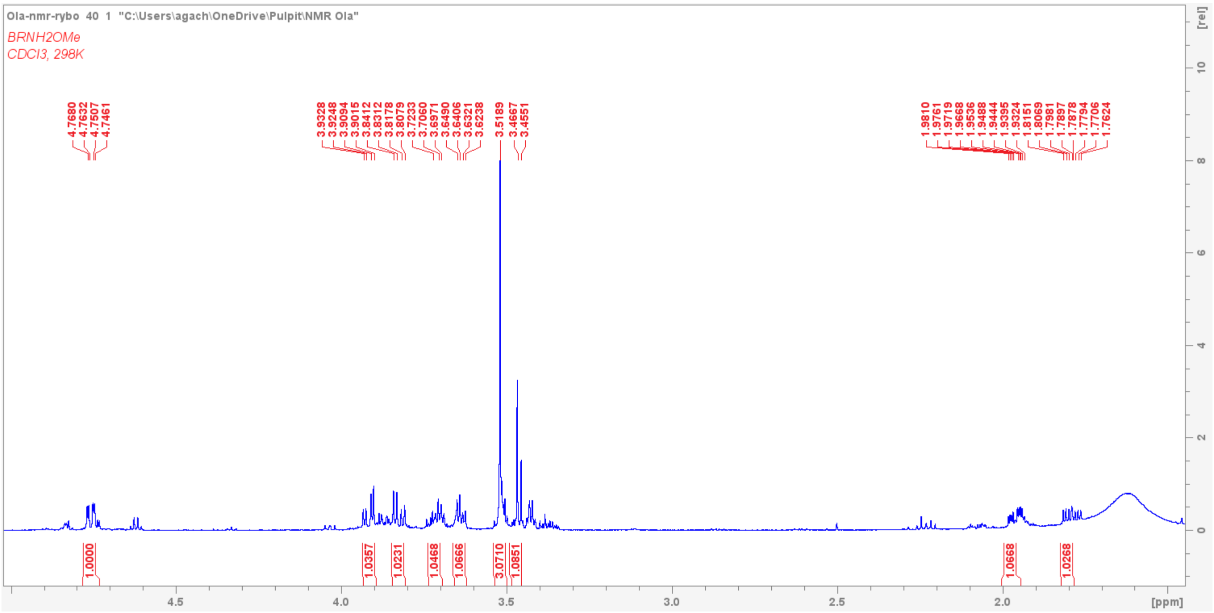

Figure S11. ^{1}H NMR spectrum of the compound **BRNH$_2$OMe** registered in $CDCl_3$; 298 K.

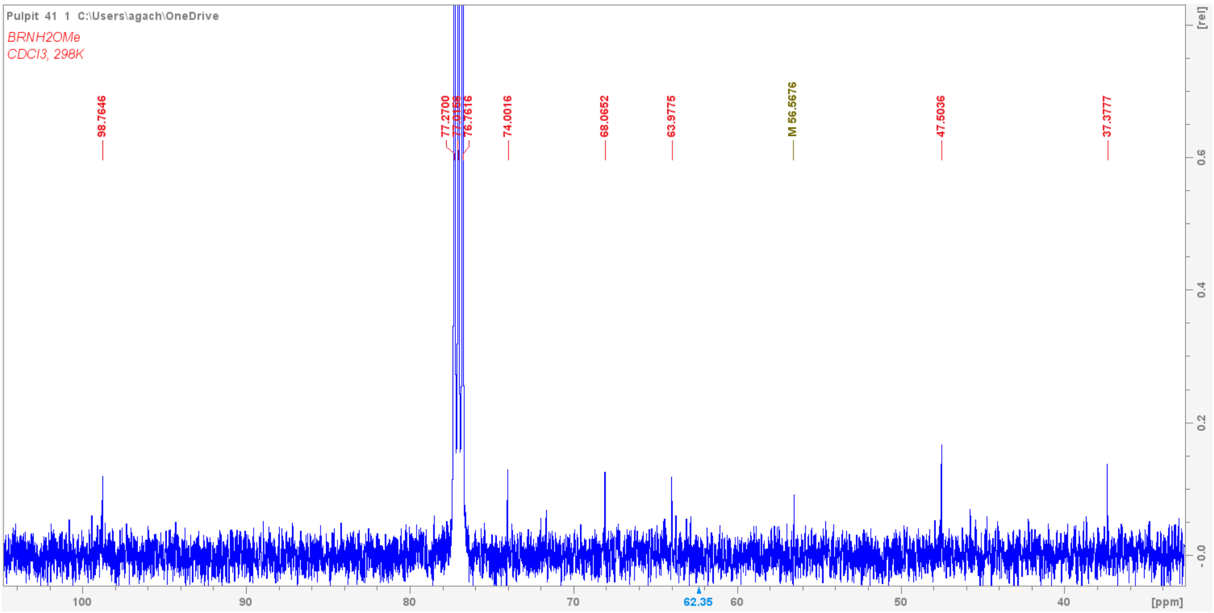

Figure S12. ^{13}C NMR spectrum of the compound **BRNH$_2$OMe** registered in $CDCl_3$; 298 K.

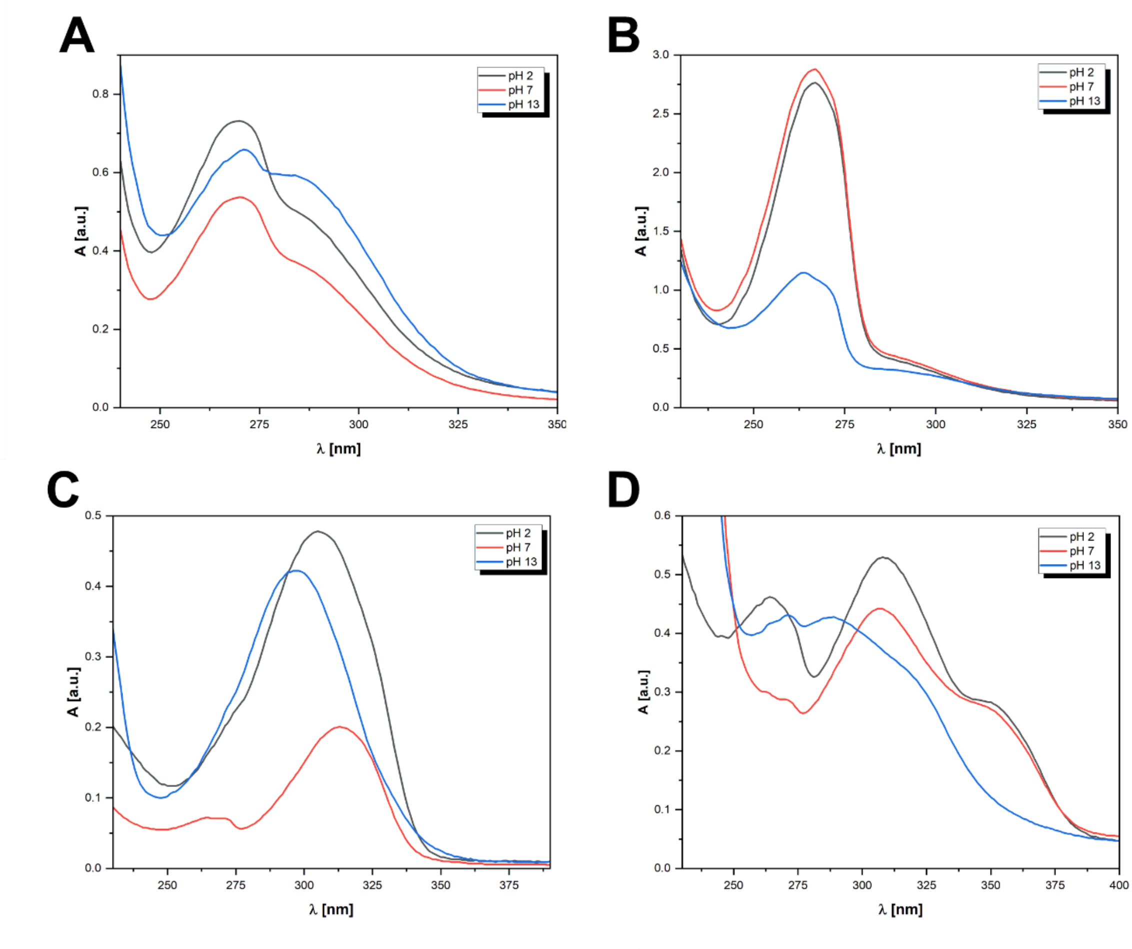

Figure S13. Absorption spectra of the **ARN$_3$OMe** (**A**), **BRN$_3$OMe** (**B**), **ARNH$_2$OMe** (**C**) and B**RNH$_2$OMe** (**D**) in aqueous (black line), acidic (red line) and basic (blue line) solutions with the same concentrations.

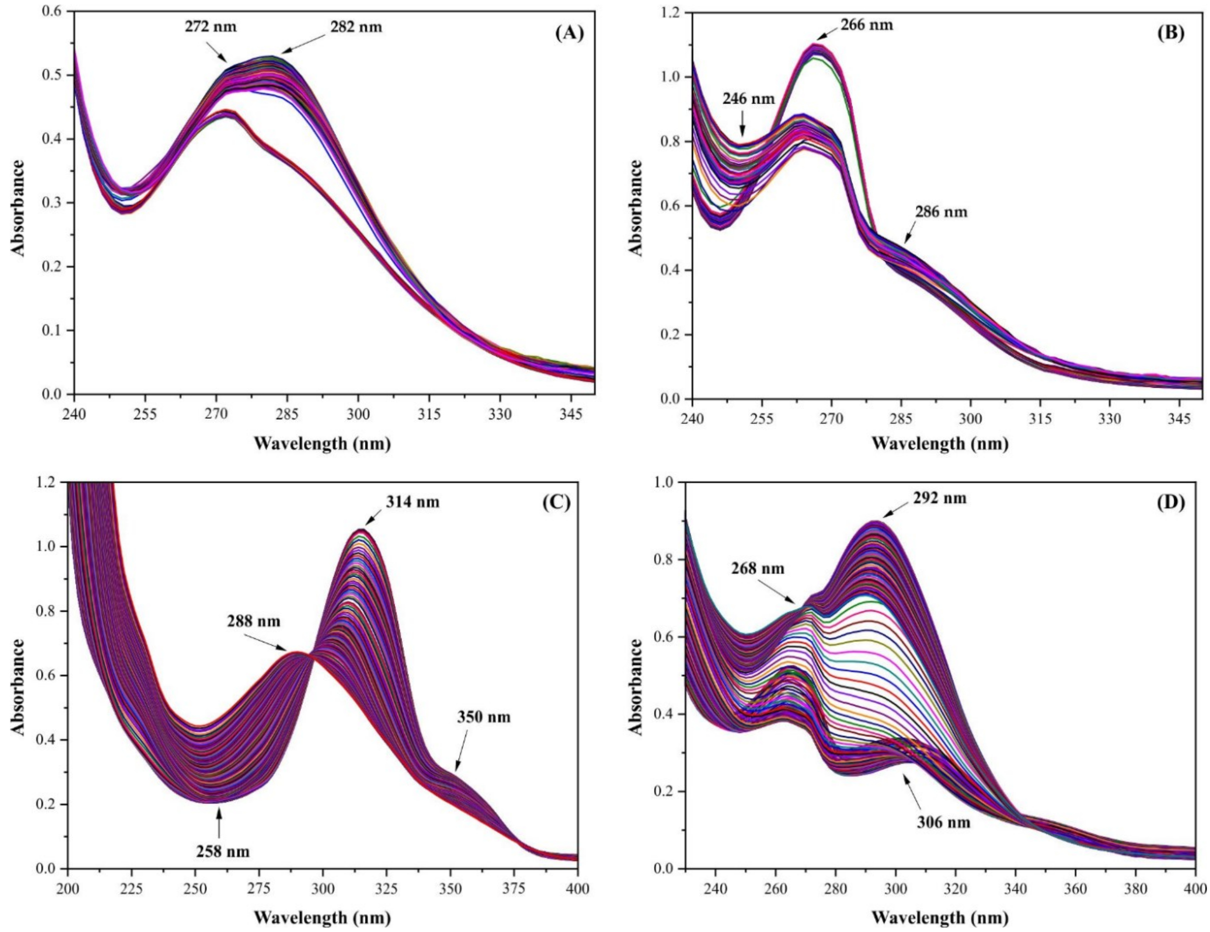

Figure S14. Changes occurring in absorption spectra of solution of investigated compounds: **A.** ARN_3OMe; **B.** BRN_3OMe; **C.** $ARNH_2OMe$, and **D.** $BRNH_2OMe$ during titration.

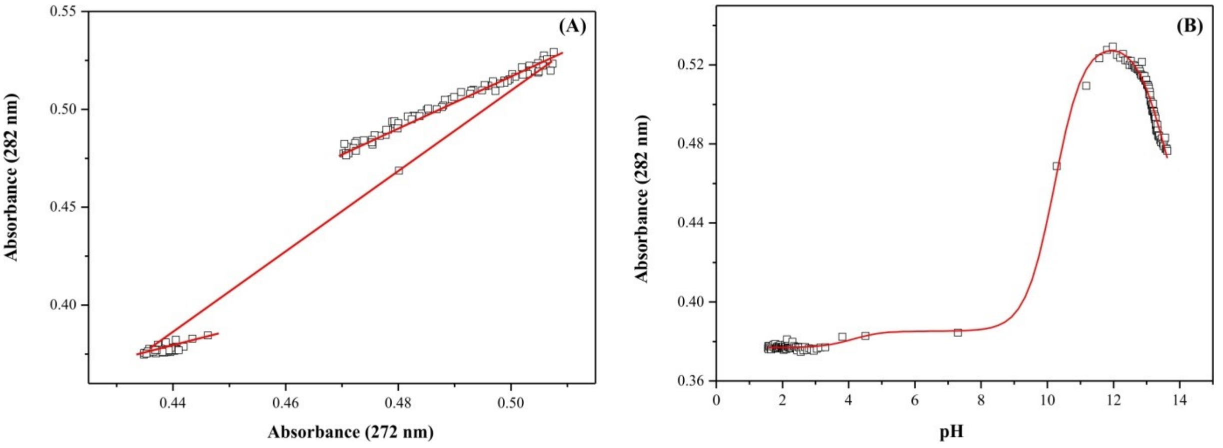

Figure S15. Plot of absorbance at 272 nm *vs*. absorbance at 282 nm (**A**); fitting red line and experimental points of absorption *vs* pH at a selected wavelength as the result of pH-spectrophotometric titration (**B**) for $\mathbf{ARN_3OMe}$.

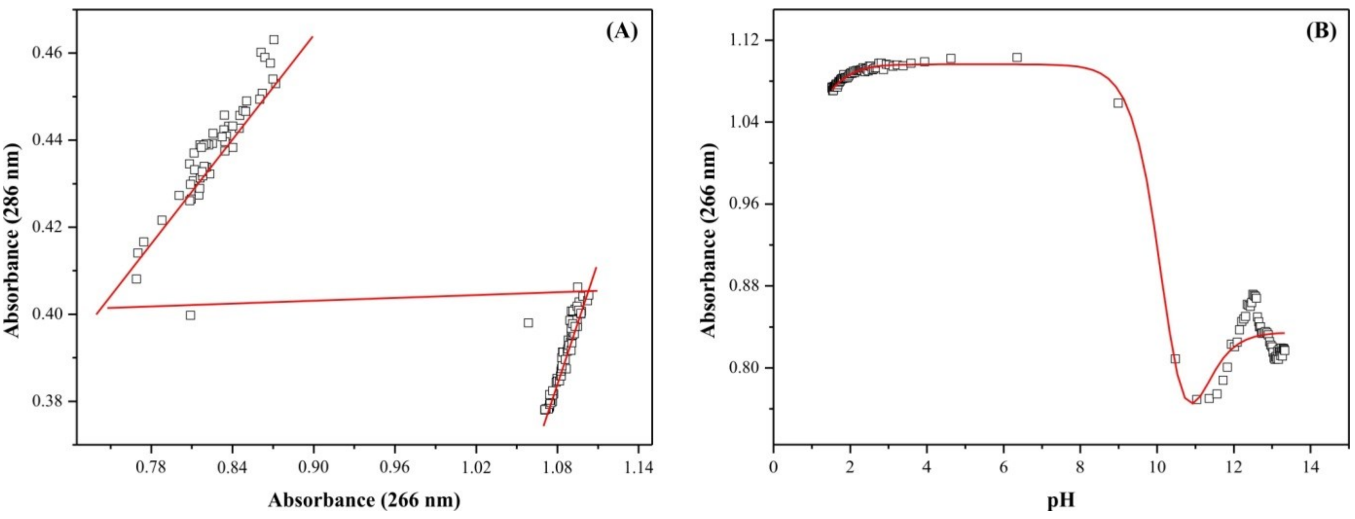

Figure S16. Plot of absorbance at 266 nm *vs*. absorbance at 286 nm (**A**); fitting red line and experimental points of absorption *vs* pH at a selected wavelength as the result of pH-spectrophotometric titration (**B**) for **BRN$_3$OMe**.

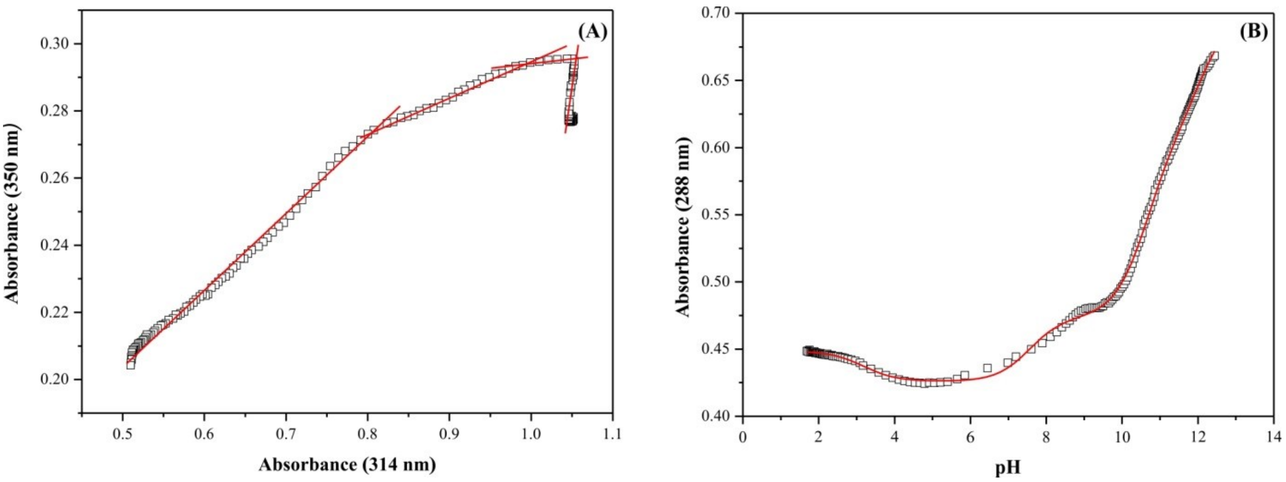

Figure S17. Plot of absorbance at 314 nm *vs*. absorbance at 350 nm (**A**); fitting red line and experimental points of absorption *vs* pH at a selected wavelength as the result of pH-spectrophotometric titration (**B**) for **ARNH$_2$OMe**.

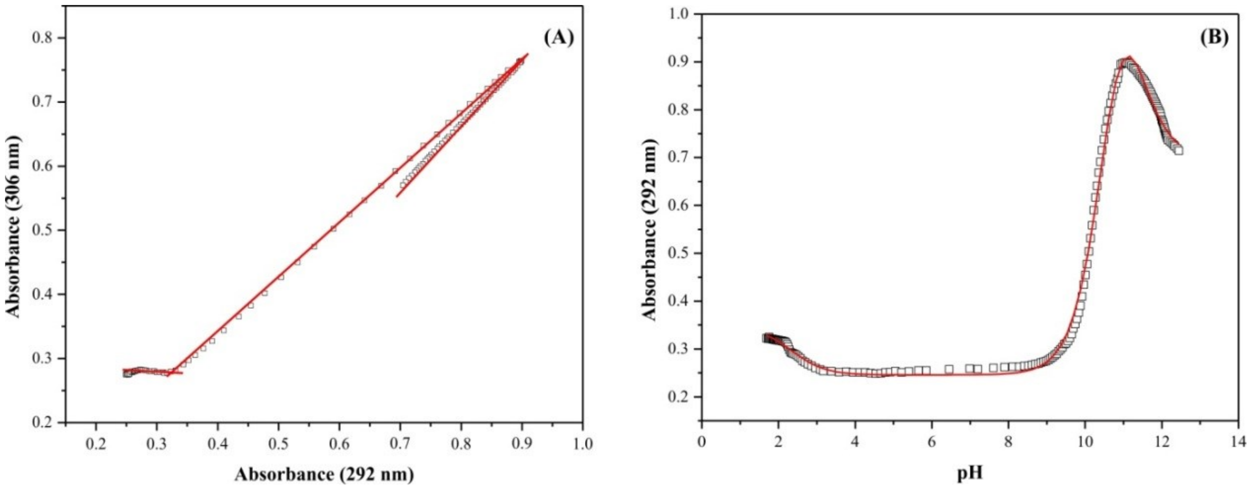

Figure S18. Plot of absorbance at 292 nm *vs*. absorbance at 306 nm (**A**); fitting red line and experimental points of absorption *vs* pH at a selected wavelength as the result of pH-spectrophotometric titration (**B**) for **BRNH$_2$OMe**.

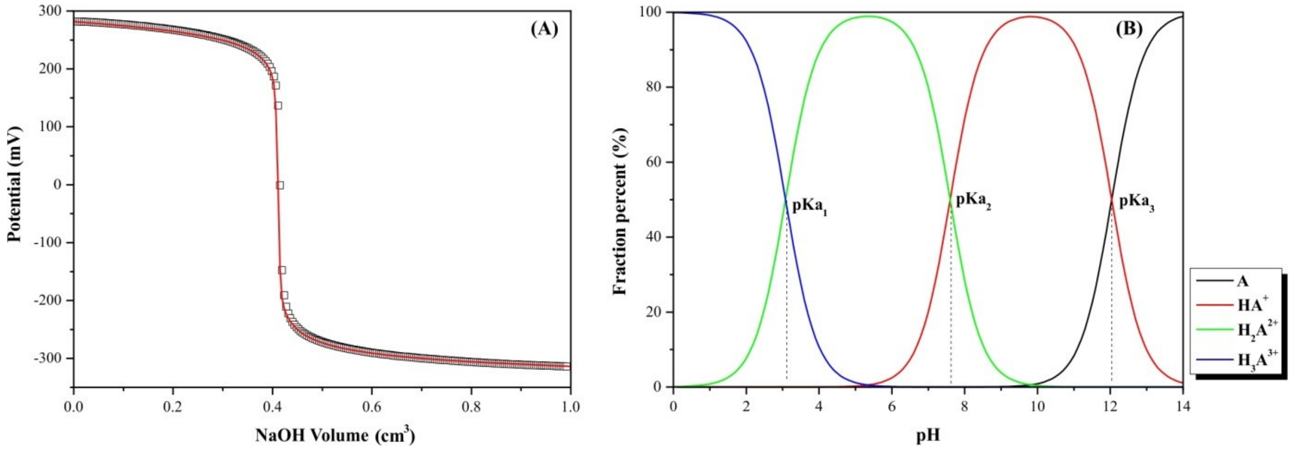

Figure S19. Potentiometric titration curve – experimental points and fitting red line (**A**) and corresponding microspecies distribution (%) diagram at the given pH (**B**) for **ARN$_3$OMe**.

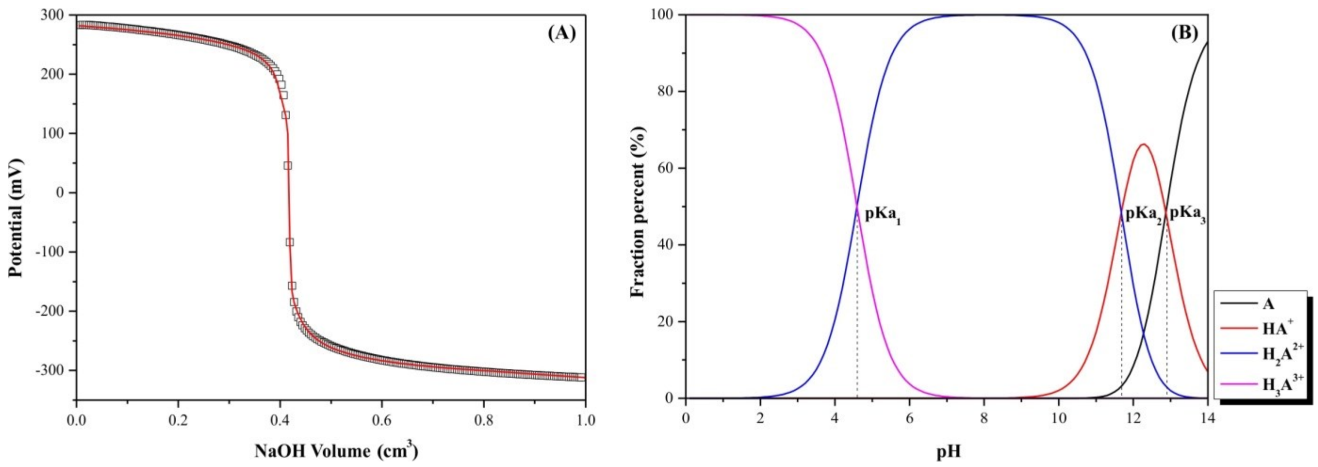

Figure S20. Potentiometric titration curve – experimental points and fitting red line (**A**) and corresponding microspecies distribution (%) diagram at the given pH (**B**) for **BRN$_3$OMe**.

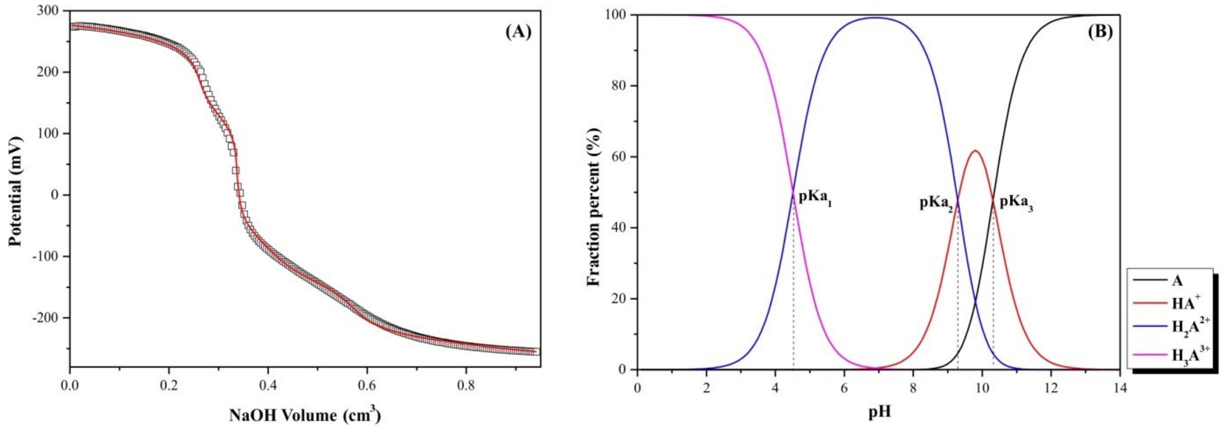

Figure S21. Potentiometric titration curve – experimental points and fitting red line (**A**) and corresponding microspecies distribution (%) diagram at the given pH (**B**) for **ARNH$_2$OMe**.

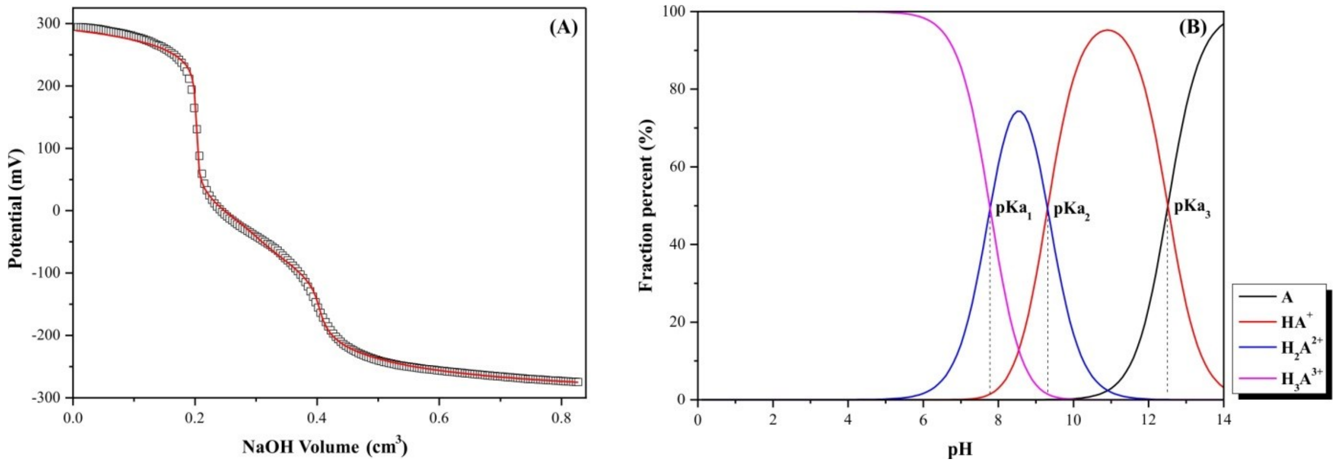

Figure S22. Potentiometric titration curve – experimental points and fitting red line (**A**) and corresponding microspecies distribution (%) diagram at the given pH (**B**) for **BRNH$_2$OMe**.

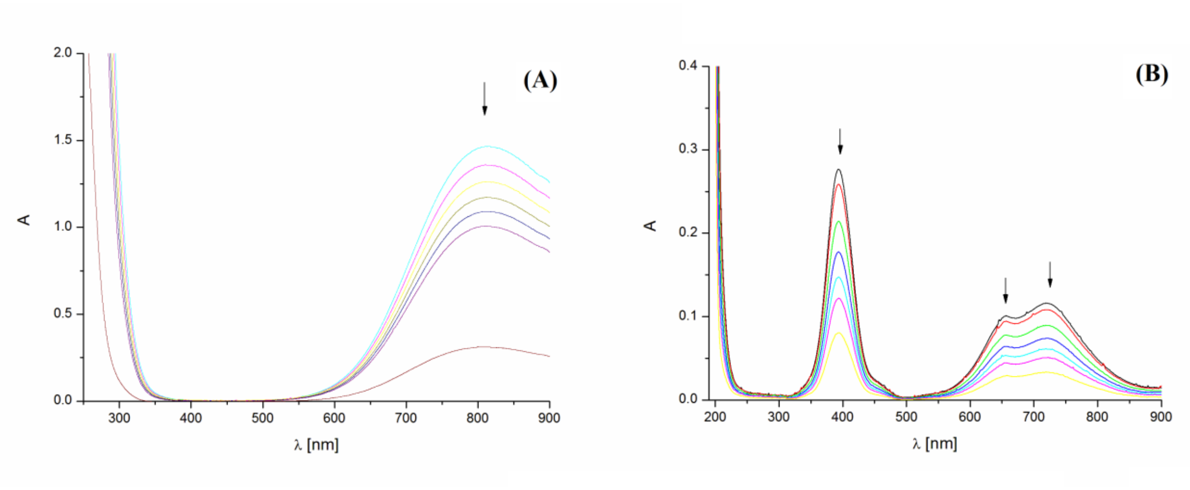

Figure S23. Gradual changes of absorption spectra of copper(II) chloride (**A**) and nickel(II) chloride (**B**) in water solutions with different concentrations.

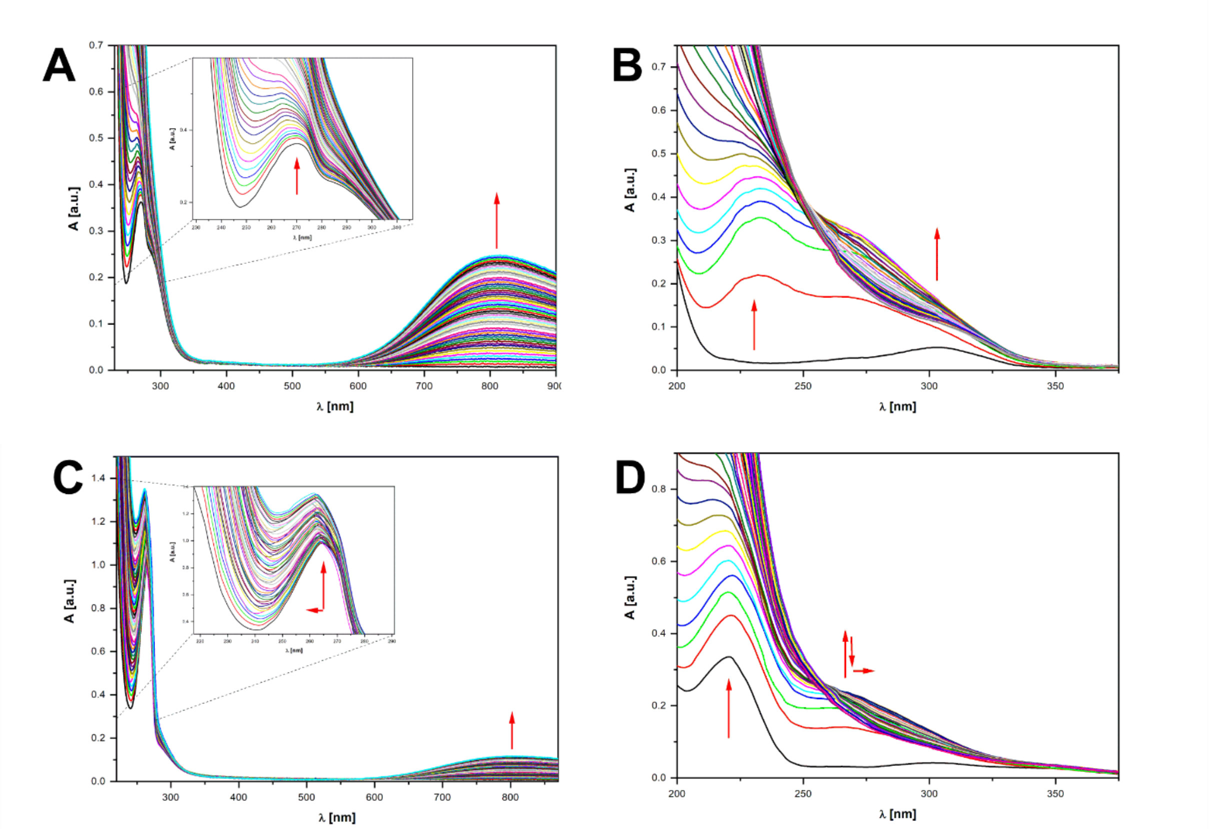

Figure S24*.* The changes in the electronic absorption spectra during the complexation of **A.** ARN$_3$OMe, **B.** BRN$_3$OMe, **C.** ARNH$_2$OMe, **D.** BRNH$_2$OMe with $CuCl_2$ in water, 298 K.

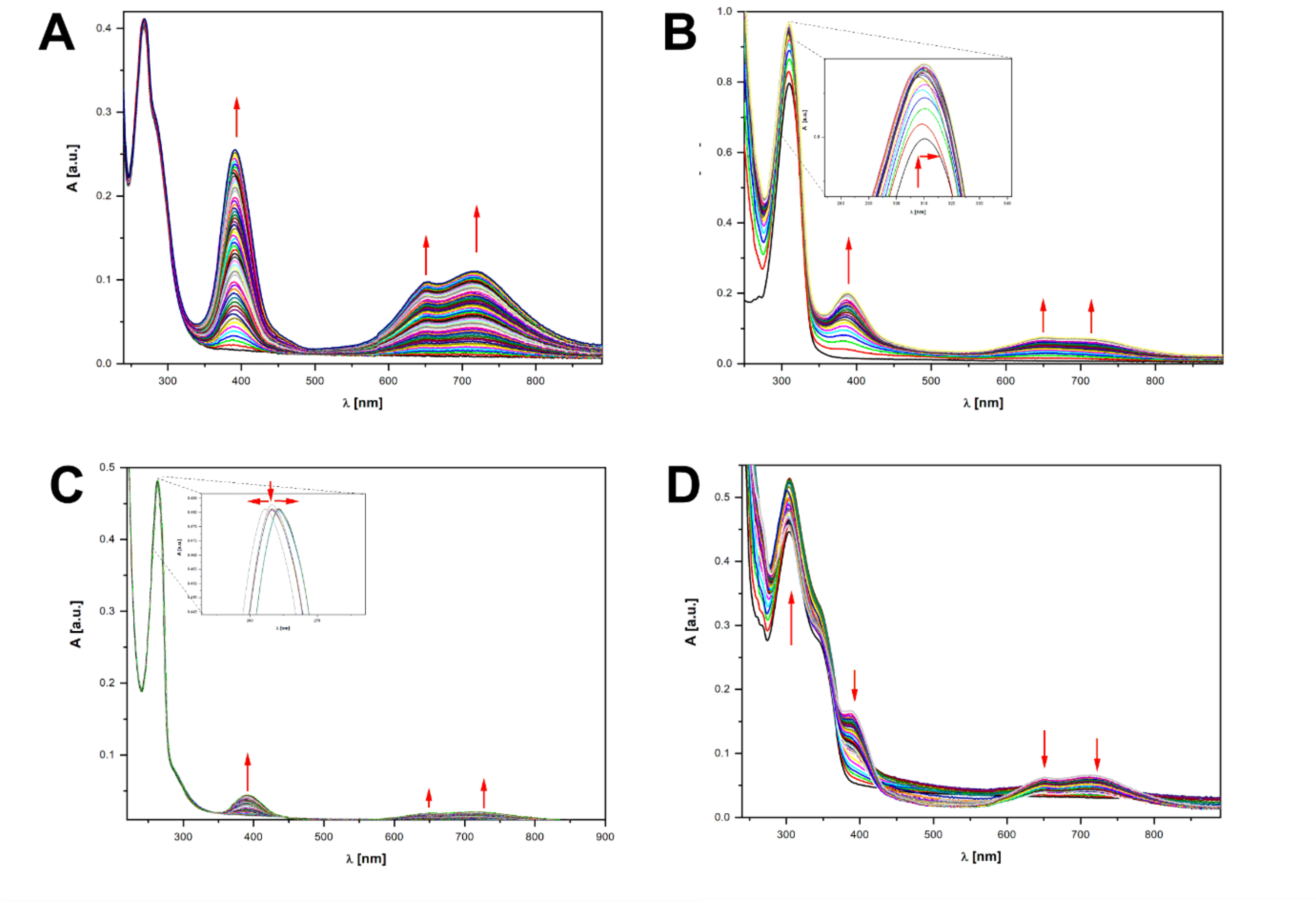

Figure S25. The changes in the electronic absorption spectra during the complexation of **A.** ARN$_3$OMe, **B.** BRN$_3$OMe, **C.** ARNH$_2$OMe, **D.** BRNH$_2$OMe with $NiCl_2$ in water, 298 K.

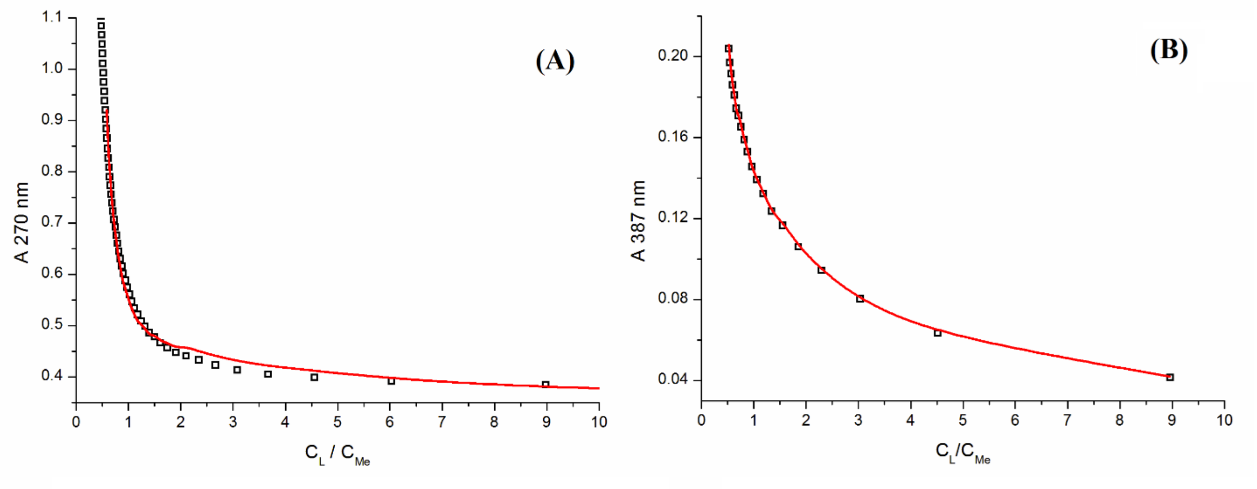

Figure S26. Selected computer fit of absorbance-mole ratio data obtained from the complexation of Ni(II) ions with compounds **ARN$_3$OMe** and **BRN$_3$OMe** (**A**) and Ni(II) ions with compounds **ARNH$_2$OMe** and **BRNH$_2$OMe** (**B**) at 25 °C. Experimental (black points) and calculated (red line) model for the 2° equilibrium (**Table 4**)

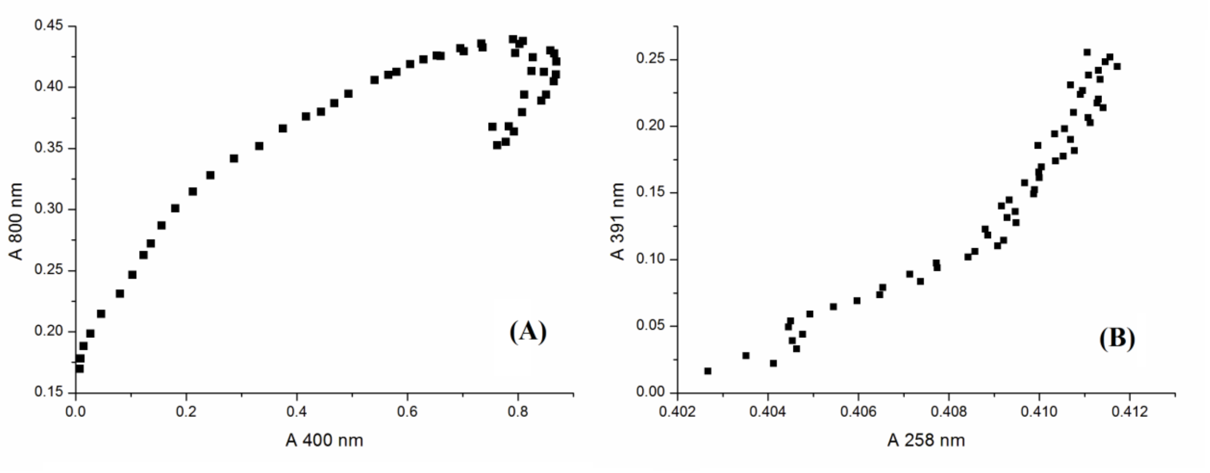

Figure S27. Selected A-diagrams were obtained as a result of titration of **ARNH₂OMe** with Cu(II) (**A**) and **ARN₃OMe** with Ni(II) (**B**).

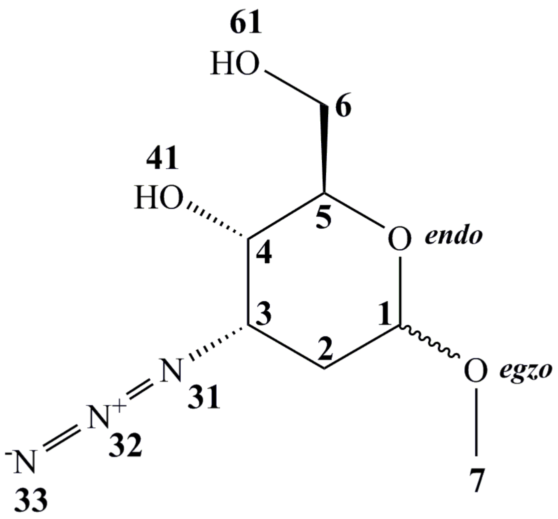

ARN_3OMe (1)
BRN_3OMe (2)

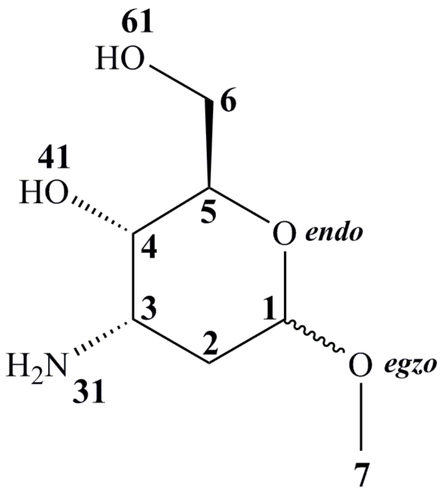

$ARNH_2OMe$ (3)
$BRNH_2OMe$ (4)

Figure S28. Schematic structures with the atom numbering of all compounds with the atom numbering (attached to **Table 5**).
